# Understanding the game experiences and mental health of youth: protocol for the Game-in-Action Quebec cohort study

**DOI:** 10.1136/bmjopen-2025-103685

**Published:** 2025-09-01

**Authors:** Vincent Paquin, Ian Raugh, Megan Davies, Martin Lepage, Katie M. Lavigne, Jacinthe Dion, Amal Abdel-Baki, Jai L. Shah, Srividya N. Iyer, Manuela Ferrari

**Affiliations:** 1 Department of Psychiatry, McGill University, Montreal, Quebec, Canada; 2 Lady Davis Institute for Medical Research, Jewish General Hospital, Montreal, Quebec, Canada; 3 Douglas Mental Health University Institute, Montreal, Quebec, Canada; 4 Département de psychologie, Université du Québec à Trois-Rivières, Trois-Rivières, Quebec, Canada; 5 Département de psychiatrie, Université de Montréal, Montreal, Quebec, Canada; 6 Centre hospitalier de l’Université de Montréal, Montreal, Quebec, Canada

**Keywords:** Digital Technology, MENTAL HEALTH, Behavior, Social Interaction, Social Support, Internet

## Abstract

**Abstract:**

**Introduction:**

Video games have been linked to a range of positive and negative effects on the mental health of adolescents and young adults. However, to better understand how games affect the mental health of young people, their use and experiences must be situated in the sociocultural and personal life contexts of individuals. Drawing from a cultural-ecosocial approach, this study combines cross-sectional and digital phenotyping measures to examine the effects of video games on the mental health of youth.

**Methods and analysis:**

Participants will be young people aged 16–25 years from the community and living in the province of Quebec, Canada. An initial sample of 1000 youth will complete a cross-sectional survey online, including measures of socio-demographic context, gaming practices and experiences, streaming practices and experiences, as well as personality and well-being. Qualitative questions will explore personal views on games and mental health. A subsample of 100 participants will be selected for digital phenotyping, including daily surveys of well-being, gaming, streaming and social experiences, combined with passive mobile sensing (eg, geolocation). Analyses will include regression and mixed models for quantitative data, reflexive thematic analysis for qualitative data, and an integration of quantitative and qualitative results using participatory methods.

**Ethics and dissemination:**

The study received ethical approval from the Institutional Review Board of McGill University (24-02-015). The dissemination of results will be conducted in partnership with a multi-stakeholder advisory committee, including youth who play video games, and will involve peer-reviewed publications, presentations to policymakers in Quebec, and workshops for clinicians and researchers.

**STRENGTHS AND LIMITATIONS OF THIS STUDY**

- The inclusion of structural factors, social affiliation through play and streaming-related experiences addresses gaps in the literature regarding the sociocultural influences of games on mental health.
- Digital phenotyping will allow for the robust examination of within-person dynamics that are independent from the confounding influences of unmeasured individual traits.
- The combination of quantitative and qualitative data will provide a more comprehensive and nuanced understanding of games and mental health.
- Convenience sampling in a specific region (the Quebec province in Canada) limits the generalisability of the results.
- Given the heterogeneity of game genres and social contexts surrounding game experiences, the study measures can only partially capture their diverse ramifications for mental health.

## Introduction

 Video games are highly popular among youth aged 16–25 years old.^1 2^ In the province of Quebec, Canada, the Entertainment Software Association of Canada reports that 71% of young adults own a video game console, and 41% of Quebecers (10% more than other Canadians) play games regularly.^3^ In Canada, the average video gaming time is 6.5 hours/week among female young adults and 10.2 hours/week among male young adults, in line with global trends.^4 5^ Some 82% of male and 58% of female players declare themselves to be ‘gamers’.^6^ For the 74% of Canadians who grew up playing video games, 74% are passing on the hobby, playing video games with their children as a way of spending time together.

Although video gaming has become a multigenerational activity, there is a long history of concerns about its potential negative impacts on the well-being of young people. Early studies have suggested that violent games increase the aggressivity of young players,^7^ or that excessive screen time might be at the expense of sleep, school, work, physical activity or interpersonal activities necessary for maintaining a person’s overall functioning and well-being.^8 9^ Screen time refers to the amount of time spent on electronic devices, such as video games, and is commonly perceived as a behaviour that can negatively influence physical and mental well-being.^10^ These putative effects of video games and screen time have become controversial owing to methodological and conceptual issues (a review of which is beyond the scope of this protocol), but they continue to animate scientific and popular discourses on video games.^11^

Another concern about games is that they may become the source of addictive behaviours. The International Classification of Diseases, 11th revision, recognised this addictive potential under the label of gaming disorder, defined as ‘impaired control over gaming, increasing priority given to gaming over other activities (…), and continuation or escalation of gaming despite the occurrence of negative consequences’.^12^ Critical perspectives on gaming disorder have highlighted the challenge of differentiating high engagement in video games that is not pathological from ‘truly’ addictive forms of gaming.^13 14^ Clinical research seeks to advance the detection and treatment of gaming disorder with the hope of improving youth mental health,^15^ and in this context, it has been suggested that high engagement in games might trigger or amplify psychotic symptoms in some individuals.^16^ However, developmental research into gaming and psychotic-like experiences suggests that the two may be linked through shared risk factors, such as challenging life circumstances, rather than a causal effect of gaming on psychosis.^17 18^

While concerns about problematic gaming have dominated clinical discourse, there is evidence for positive effects of video gaming, which may help explain why some people turn to video games to deal with challenging situations. Observational research using in-the-moment (ie, ambulatory) assessments and natural experiments has shown that people’s mood improves when they play video games,^19 20^ suggesting a role for video gaming as an emotion regulation strategy. The experience of immersing oneself in games may contribute to providing a relief from day-to-day stressors while providing an alternative environment to fulfil psychological needs (eg, of social connection).^21^ Surveys, laboratory experiments, qualitative interviews and ethnographic research support the importance of social connectedness enabled by video games for well-being.^22^,^25^ Moreover, games afford opportunities to embody virtual characters with desirable traits or elevated social status, conferring opportunities for young people to explore and adaptively experiment with their identity.^22 26^ Finally, some video games solicit cognitive functions in ways that may enhance processing, memory and multitasking skills over time.^27^

Beyond the immediate context of game play, shared interest in games serves as a scaffolding for communities of gamers who meet and socialise in a variety of spaces. Fan communities of video games may enrich the social capital of young players by offering opportunities to establish new relationships and make a living from streaming activities;^28^ however, they may also be places of exclusion, harassment and discrimination that hamper the well-being of their members.^29^ Streaming platforms such as Twitch are key spaces where people interact over a common interest in a game, a game performance and/or a content creator (the streamer).^30^ The culture of esports (competitive and/or professional game play) and streaming expands the range of experiences enabled by video games: through these game-related productions and events, people interested in video games can interact with like-minded peers (eg, by contributing to group discussions in streaming channels) and engage with the narratives and mechanics of play without being the player. Although the popularity of video game streaming has significantly increased over the last decade, the role of this phenomenon in the mental health of young gamers remains underexplored in research.

## Theoretical framework

The range of putative benefits and harms of video games for mental health is not surprising when we consider the diversity of games, the psychological features and personal histories of players, and the sociocultural contexts in which gamers and games are embedded.^31 32^ We posit that a cultural-ecosocial approach to mental health^33^ provides a helpful framework for examining the multifaceted connections between games and mental health. According to the cultural-ecosocial view, environmental influences on mental health can be disaggregated into at least four domains: (1) Lifespan developmental history, (2) Social structure and positioning, (3) Cultural meaning, norms, values and affordances and (4) Individual biography and self-understanding. This approach builds on previous biopsychosocial accounts of mental health by specifying the mechanisms that connect environment, culture and mental health^34^ and has been proposed as a framework for advancing neuroscience and precision psychiatry research.^35 36^

*Lifespan developmental history*. Lifespan development encompasses the psychological needs, social roles and unique psychological traits of individuals that shape the impacts of games on mental health. Granic^26^ argued that for adolescents and young adults, games offer rich opportunities to develop their identities, practise prosocial behaviours and form intimate bonds with their peers, which are central to this period of life. Self-determination theory advances that gaming can be motivated by the fulfilment of psychological needs for autonomy, competence and connectedness,^25^ and empirical evidence suggests a positive effect on the well-being of players who successively satisfy these needs through video games.^37^ However, excessive video gaming (ie, gaming disorder) may interfere with role fulfilment, including relationships with peers and family, as well as school functioning, for some individuals.^38^ Digital phenotyping, which involves ambulatory collection of active (eg, surveys) and passive (eg, geolocation) data, may provide indirect markers of gaming disorder;^39^ notably, greater time spent at home according to geolocation has been associated with depressive symptoms^40^ and could hypothetically index reduced mobility as a consequence of prolonged gaming sessions. Importantly, the practices and outcomes of video games vary in part as a function of the psychological propensities of players. For example, there is some evidence that the experience of being immersed in a video game (ie, the degree to which the person feels absorbed in the game^41^) is associated with improvements in emotional states,^42^ but immersion has also been hypothesised to contribute to the risk of gaming disorder.^43^ A better understanding of how individuals fulfil their developmental needs and self-determination through games, and how they differ in their propensity to experience immersion, could support a more personalised assessment of the risks and benefits of games.

*Social structure and positioning*. Social structures and the positioning of individuals can influence how and why they engage with games. In the face of social exclusion or challenging life circumstances, some people will turn to video games as a means of immersing themselves into a more welcoming environment and for making friends online.^21 24 44^ For individuals living in socioeconomic precarity, games may be a safer and more accessible activity relative to other pursuits, which could explain why lower household income or parental socioeconomic status are associated with higher levels of adolescent video gaming in some samples.^18 45^ Disability may increase the salience of gaming as an activity that is relatively more accessible than others to fulfil psychological needs for self-determination;^46 47^ however, disability may also limit the accessibility of games that solicit particular physical or cognitive resources. Women, queer and racialised gamers are at increased risk of exposure to harassment and discrimination in game communities and online multiplayer games.^48 49^ These examples illustrate how social structures and positioning can predispose to or interfere with a range of game-related experiences.

*Cultural meaning, norms, values and affordances*. The shared norms and values of a person’s social network may encourage or discourage certain ways of engaging with games. For one, being part of a group of friends who share one’s interest in games lends a particular meaning to games as something that fosters and nourishes friendships. Playing games jointly with friends or family affords an opportunity for social connection that contributes to individual well-being.^50^ In the absence of such social ties, there may be fewer opportunities to integrate in-game accomplishments and experiences outside of the game environment—and possibly increased risk of facing the disapproval of others for what is often deemed to be excessive ‘screen time’.^51^ Although the role of these cultural factors in the mental health effects of games remains underexplored, we can posit benefits (eg, social connectedness) and risks (eg, stigma and isolation) related therefrom. Positive and negative effects of games may thus be uncovered in research by considering the alignment or mismatch of gaming behaviours with norms and values in the person’s social environment.

*Individual biography and self-understanding*. Unique aspects of gaming experiences might influence the self-concept and well-being of individuals in ways that are not fully captured by existing theoretical frameworks. Focusing solely on theorised mechanisms of effects on mental health, such as gaming disorder or psychological need satisfaction, risks overlooking the unique ways in which games can bring pivotal life experiences for individuals.^26 52^ For this reason, exploring the lived experiences of young players in an open-ended fashion, for example, through qualitative and participatory methods, is essential for producing a comprehensive understanding of gaming and mental health.

## The present study

Research published to date has largely relied on cross-sectional samples, limiting the capacity to remove confounding influences from individual characteristics of participants. In addition, existing research infrequently accounts for streaming practices and the role of social factors in the associations between video games and mental health. As highlighted above, video games may have different impacts on mood and well-being depending on the person’s social context, the psychological mechanisms that are activated and the overall characteristics of video game habits and streaming activities. Given the potential influences of social context on these associations, it is necessary to consider features such as socio-economic status, gender, ethnoracial identity, (dis)ability, as well as game-related practices that are shared with friends or family.

**Figure 1:**
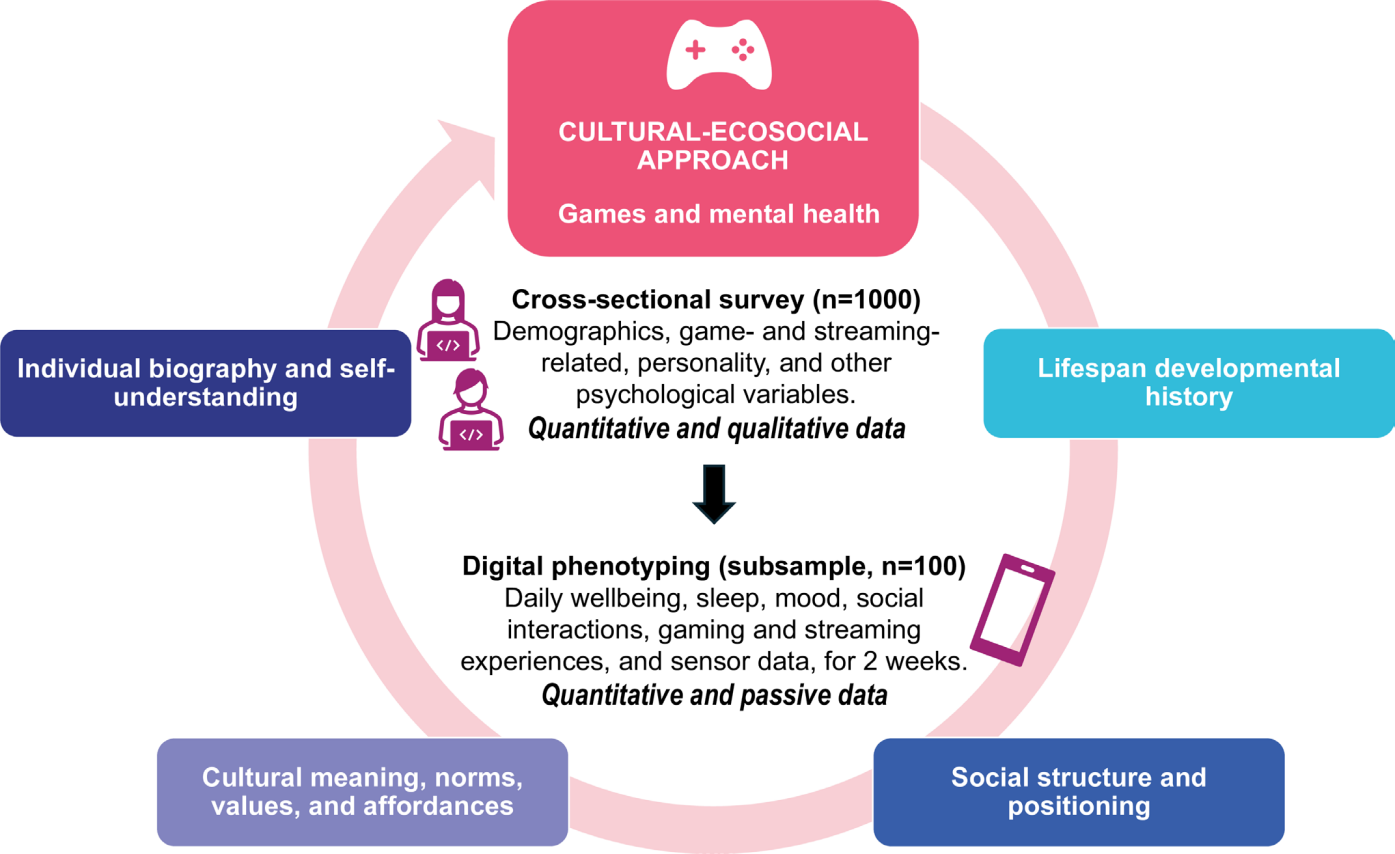
Summary of the study design and theoretical framework.

This study will thus aim to investigate the associations of video game-related experiences with mental health in youth aged 16–25 years old from the general population of the province of Quebec, Canada. The study will have two components: a cross-sectional survey of 1000 individuals recruited by convenience in the community and including a mix of quantitative and qualitative items, followed by digital phenotyping (ie, daily diaries and mobile sensing) in a subsample of 100 individuals over 2 weeks. The key objectives of the study, listed in table 1, focus on psychological needs, immersion, game-related social affiliation, harassment and discrimination, and self-narratives about games and mental health. A distinctive feature here is the inclusion of psychotic-like experiences, which are rarely investigated in the field of games and media effects but are considered a transdiagnostic marker of risk for mental health problems during adolescence and early adulthood.^53^ Additional objectives will be to examine the associations of streaming-related experiences with well-being, and to explore the predictive utility of mobile sensing data (eg, geolocation). This study follows the Strengthening the Reporting of Observational Studies in Epidemiology reporting guideline.^54^ Figure 1 summarises the study design and theoretical framework.

**Table 1:** Main objectives of the study and related variables

Predictors	Outcomes	Framework domains
*Objective 1. To examine whether the satisfaction of psychological needs and social affiliation through games is associated with well-being* Hypothesis: Greater play time with others, higher scores for belonging and need satisfaction, and lower scores for need frustration will be associated with lower levels of psychotic-like experiences and higher levels of well-being		
Play time with others (C) Fandom Functions Scale—Belonging (C) Basic Needs in Games Scale, gaming and streaming (L)	Psychotic-like experiences (C) Daily well-being questionnaire (L)	Lifespan developmental history; Cultural meaning, norms, values and affordances
*Objective 2. To examine whether a greater propensity for immersion is associated with better satisfaction of psychological needs and improved well-being through games* Hypothesis: Higher scores for absorption and daily immersion will be associated with higher levels of fandom functions, need satisfaction and daily well-being, but also higher levels of psychotic-like experiences and problematic gaming		
Tellegen Absorption Scale (C) Daily immersion (L)	Fandom Functions Scale (C) Basic Needs in Games cale, gaming and streaming (L) Daily well-being (L) Psychotic-like experiences (C) Problematic gaming (C)	Lifespan developmental history
*Objective 3. To examine the associations of marginalised identities with exposure to harassment and discrimination in games or streaming, and the mediated associations with well-being* Hypothesis: Participants identifying as female, transgender or non-binary, from an ethnoracial minority, or having a history of mental health problems will report higher levels of exposure to bullying, which will mediate an association with lower levels of daily well-being		
Sex and gender (C) (Dis)ability (C) Racialised identity (C) History of mental health problems (C)	*Mediator:* Exposure to bullying (C, L) Daily well-being (L)	Social structures and positioning
*Objective 4. To explore young people’s narratives about the role of games and streaming in their lives and their mental health* This exploratory objective has no hypothesis and will rely on qualitative data from open-ended questions (C)		
N/A	N/A	Individual biography and self-understanding

C, cross-sectional sample (n=1000); L, longitudinal, digital phenotyping subsample (n=100).

## Methods and analysis

### Study population

This study will include a cross-sectional questionnaire followed by a 2-week digital phenotyping component in a subsample. For the cross-sectional questionnaire, we will recruit 1000 adolescent and adult gamers from the general population in the province of Quebec, Canada. Eligibility criteria will include: (1) Age 16–25 years, (2) Playing video games at least 1 hour per week and (3) Speaking English or French. Of this sample, 100 participants will be selected to participate in the digital phenotyping component; these participants will be randomly selected after stratification according to their gender (to ensure at least 30% of cisgender boys or men, at least 30% of cisgender girls or women and at least 10% of transgender or non-binary individuals). Digital phenotyping will include daily surveys (sometimes referred to as ecological momentary assessment or experience sampling^55^) and mobile sensing. The daily surveys and mobile sensing data will be collected for 2 weeks using the Beiwe application installed on participants’ smartphones.

### Rationale for the sample size

Given the variety of objectives and analyses planned, we established the sample size based on two basic scenarios: a linear regression of one cross-sectional variable over another (n=1000) and a generalised linear mixed model where one daily digital phenotyping variable is regressed over another (n=100). For the cross-sectional scenario, we conducted the power analyses in G*Power, V.3.1.9.7. We calculated that a sample of n=1000 will be sufficient to detect a linear regression slope of β=0.09, assuming a type I error rate of 0.05 (two-tailed) and statistical power of 85%. This would be sufficient to evaluate, for example, a weak but significant association between greater belonging and lower levels of psychotic-like experiences (Objective 1) or a greater propensity for immersion and greater psychological functions of games (Objective 2). For the digital phenotyping component, we used the sample size calculator developed by Barnett and colleagues:^56^ https://onnela-lab.shinyapps.io/digital_phenotyping_sample_size_calculator/. We found that a sample size of 100 persons with daily measures over 2 weeks will provide statistical power of 93% to detect effect sizes of β=0.07 with a type I error rate of 0.05, assuming 50% missing data and random-effect variance of 1.1. These parameters would be sufficient to examine the association between daily needs satisfaction in games and daily well-being (Objective 1) or the association between daily immersion and daily well-being (Objective 2). This statistical power estimation also applies to passive sensor data (eg, geolocation) transformed into daily features (eg, daily time spent at home).

### Procedures for recruitment, data collection and retention

Recruitment for the cross-sectional study started on 15 September 2024 and is ongoing. Participants are recruited through advertisements on social media targeting the age range and geographic region of interest. To maximise sample diversity, we have contacted several community organisations with missions related to mental health, LGBTQ+communities, and the well-being of immigrant and racialised youths, as well as university student groups and online communities with game-adjacent interests (eg, roleplay games, cosplay, geek) across the province of Quebec who circulated the advertisement through their networks. Recruitment will stop after achieving the targeted sample size. After being directed to a website presenting the study, individuals will be screened for eligibility and will provide virtual consent to participate in the cross-sectional questionnaire via electronic signature. Participants will then complete the cross-sectional questionnaire on REDCap, after which they will be invited to complete additional measures (cognitive tasks) on PsychoJS, which will be described in a future publication. Participants who provide optional consent to complete the cognitive tasks on PsychoJS will receive a link by email at a later time.

As part of the cross-sectional questionnaire, participants will be asked if they agree to be contacted for the digital phenotyping component. Participants of the digital phenotyping component will be randomly selected by email or phone from the list of responders who expressed their interest and will be invited to a 30 min virtual individual meeting with a research assistant. During the meeting, the research assistant will present the study protocol and consent form for the digital phenotyping. Consent will be provided by electronic signature. Then, participants will receive assistance to install the Beiwe application on their smartphone.

To compensate participants for their time, they will be offered $35 in gift cards for participating in the cross-sectional survey. Participants in the digital phenotyping will receive $5 for each daily questionnaire completed (ie, a total of $70 if all questionnaires are completed). Throughout the follow-up period for digital phenotyping, a research assistant will be available to provide technical support as needed. An email will be sent to participants when they reach the fourth day of data collection to inquire about any difficulties.

### Beiwe application

Throughout the follow-up period, Beiwe will be used to collect passive digital data and to administer self-report questionnaires to participants of the digital phenotyping. This application is compatible with iOS and Android smartphones and automatically uploads the data it collects to secure cloud servers. Beiwe was developed by the Onnela Lab (Harvard University) and implemented in Montreal by Lanovaz and colleagues (Centre Axel). It can be downloaded from the Centre Axel website (https://beiwe.centreaxel2.com/download) for Android phones and from the App Store for iOS phones. Participants will be instructed to let the application run in the background throughout the 2-week follow-up period. The app will passively collect data on geolocation, accelerometry and screen activation time. Data from Beiwe will be encrypted and stored on secure servers in Quebec. Every day for 2 weeks, participants will also receive a prompt to complete a questionnaire on the application. Previous studies^39 57^ support the association of these digital markers with common mental health symptoms in population-based samples, when combined with self-report questionnaires, and will be explored here as correlates of game-related experiences and well-being.

### Cross-sectional measures

*Eligibility screening*. Questionnaires are available in the online supplemental material. Participants will report their age, year of birth, place of residence (province of Quebec: yes or no), and time spent on video games in a typical week (eight options, ranging from none to more than 20 hours per week).

*Demographic questions*. Participants will report their sex assigned at birth (male, female or intersex), gender (woman, man, non-binary, two-spirit or other), and ethnoracial identity (Arab or Middle Eastern, Asian, Black or African, Hispanic or Latinx, Indigenous, Mixed, Other or White). Participants will also report their highest level of education completed (11 options), student status (full-time, part-time or none) and employment status (full-time, part-time or none). Participants will optionally provide their postal codes for future secondary analyses of neighbourhood-related features. Lastly, participants will be asked whether they self-identify as someone with a disability (yes or no), if they have ever been diagnosed with a mental health condition, what the diagnosis was (open textbox), and if they have ever sought help or services for mental health.

*Game-related experiences and practices*. Participants will report whether they self-identify as a gamer (yes or no) and at what age they started playing games regularly. The frequency of play of 13 game genres will be reported on a scale of 1=never to 5=very often using a questionnaire adapted from Allen.^58^ Psychological functions of gaming will be assessed using a modified version of the Fandom Functions Scale,^59^ which includes 14 items evaluating the role of fan practices in providing a sense of purpose (eg, ‘Provides me with an opportunity to grow and discover more about aspects of myself’), an escape from stressors (eg, ‘Provides me with a break from life’s stresses’) and belonging (eg, ‘Provides me with a chance to expand my circle of friends’). The three subscales displayed good internal reliability (respectively α=0.93, α=0.86 and α=0.84) and were correlated with well-being and fandom identity in a previous sample of undergraduate students in the US.^59^ The opening instruction of the scale, which broadly inquired about the person’s fan interests, was modified to target participants’ ‘involvement with video games and the game community’. The use of games to recuperate from stress will be evaluated using four items adapted from the Recovery Experience Questionnaire (eg, ‘When I play video or computer games I forget about work’) rated on a scale of 1=does not apply at all to 5=does fully apply.^60 61^ The full questionnaire, tested in a sample of young adults in Germany, included four subscales (total 16 items), psychological detachment: α=0.78, relaxation: α=0.70, mastery: α=0.76, control: α=0.77, which were positively associated with greater time spent on gaming.^60^ Game play with friends, family or romantic partners, whether in person or remotely, will be reported using one item on a scale of 0=no participation to 4=at least daily, with a follow-up item inquiring about the general length of these play sessions.^61 62^ An additional, secondary measure of gaming motivations will be the Video Game Uses and Gratifications Instrument, which includes 20 items (eg, ‘I feel proud when I master an aspect of a game’) rated on a scale of 1=strongly disagree to 5=strongly agree.^63^ The questionnaire was validated in a sample of children, adolescents and university students in the US and includes six subscales (competition, challenge, social interaction, diversion, fantasy and arousal) with good internal reliability (range of α: 0.80–0.89) and positive associations with time spent playing video games. Problematic gaming will be assessed with the Gaming Disorder Test, which includes four items (eg, ‘I have difficulties controlling my gaming activity’) on a scale of 1=never to 5=very often.^64^ The scale displayed good internal reliability (α=0.84) in a sample of adult gamers in China and the UK and was positively associated with depression, loneliness and another validated measure of gaming disorder. Exposure to and perpetration of bullying in video games will be measured using three items adapted from the Olweus Bullying Questionnaire (eg, ‘How often have you been bullied while gaming in the past couple of months?’) rated on a scale of 1=never to 5=several times a week.^65^ Lastly, participants will be invited to write in open-ended textboxes about their reasons for gaming, what they like about playing video games, a time where playing games helped their mental health, a time where playing games worsened their mental health, their favourite games growing up and now, what game character they would like to be and why, and whether they feel games have an impact on their daily life and well-being.

*Streaming-related experiences and practices*. The questions in this section were modelled on those related to gaming but were adjusted to focus on watching or producing game streams. Since these modified questionnaires have not been validated, they were pilot-tested by the research team and Youth Advisory (see Patient and public involvement, below), and their psychometric properties will be examined in the present study (see Analyses, below). Participants will first be asked, ‘Do you consider yourself a streamer and/or someone who watches video game streams?’ (yes or no), and subsequent questions will only be administered if they answer ‘Yes’. Measures in this section will include streaming-focused versions of the above measures: weekly time spent on watching others play games and on streaming games, age at beginning of these activities, preferred genres (same as above), preferred platforms (eight items, eg, Twitch, YouTube, etc), a modified Fandom Functions Scale,^59^ the four items from the Recovery Experience Questionnaire,^60^ the frequency of watching or streaming with others,^62^ a modified Video Game Uses and Gratifications Instrument,^63^ a modified Gaming Disorder Test^64^ and bullying in streaming platforms.^65^ Open-ended questions will be administered regarding motivations for using streaming platforms, favourite streamers and perceived impacts on daily life and well-being.

*Personality*. Propensity for immersion will be measured with the Modified Tellegen Absorption Scale, which includes 34 items (eg, ‘The crackle and flames of a woodfire stimulate my imagination’) rated on a scale of 1=never to 5=always.^66^ This questionnaire showed good internal reliability, α=0.94 in a sample of college students in the US^67^ and has been associated with a range of traits and experiences, such as the propensity for immersion in media, hypnosis and dissociation.^68^ Personality traits of extraversion, agreeableness, conscientiousness, neuroticism and openness will be measured with the short version of the Big Five nventory, which includes 10 items (eg, ‘I see myself as someone who is reserved’) on a scale of 1=disagree strongly to 5=agree strongly, and has demonstrated good validity in adult samples in the US, Germany and France.^69 70^

*Other psychological variables*. Psychotic-like experiences will be measured with the Current Community Assessment of Psychic Experiences, which includes 15 items (eg, ‘In the past 3 months, have you felt as if the thoughts in your head are not your own?’) rated on a scale of 1=never to 4=nearly always.^71 72^ The scale displayed good internal reliability (α=0.84) and was positively associated with psychological distress in a sample aged 16–25 in Australia.^71^ Social support will be measured with the Oslo Social Support Scale, which includes three items assessing the number of people close to the person, how much interest others show in the person, and how easy it is to get help when needed.^73^ Usage in the past 2 weeks of energy drinks, alcohol, cannabis, amphetamines, non-prescribed psychostimulants and other substances will be reported on a scale of 1=never to 4=every day.^74^ Cognitive functioning will be assessed using interactive cognitive tasks on a separate platform in a subset of voluntary participants; these measures will be reported elsewhere as part of a secondary study.

### Daily surveys

*Well-being, sleep and mood*. Daily well-being will be measured with a questionnaire from the WARN-D study,^75^ which includes 10 items (eg, ‘Today, I felt down or depressed’) on a scale of 1=not at all to 7=very much. We added an additional item for somnolence (‘Today, I felt sleepy’) as a marker of sleep insufficiency. Sleep schedules will be assessed using three items from the Munich Chronotype Questionnaire (eg, ‘What time did you go to bed last night?).^76^ Affect will be assessed using a short version of the Positive and Negative Affect Schedule,^77^ with eight items (eg, ‘Today, I felt stressed’) on a scale of 1=not at all to 7=very much. In a sample of university students in the Netherlands, the scale demonstrated configural and metric invariance over different sampling frequencies and questionnaires length, and it was found to have good reliabilities for positive and negative affect at the within-person level (ω=0.818 and ω=0.815) and between-person level (ω=0.934 and ω=0.929), respectively.

*Social interactions and daily events*. Participants will report daily occurrence of in-person social interactions with two items adapted from the WARN-D questionnaire,^75^ assessing relationship categories (eg, friends, family, strangers) and the degree of enjoyment (1=not at all to 7=very much). Two more items will assess virtual social interactions following the same format. Daily events were reported using four items from the WARN-D questionnaire about the categories of most positive and negative events of the day (eg, finances, physical well-being, etc) and their intensity (1=not at all positive/negative to 7=very positive/negative).

*Gaming*. Participants will report daily instances of gaming (yes or no) and periods of the day where they played using two items adapted from Allen^58^ and Gentile *et al*.^78^ Positive social experiences (eg, receiving praise, good atmosphere) and experiences of bullying, harassment or exclusion in games will be reported on a scale of 1=never to 5=very often using 10 items from Hygen *et al* (soon to be submitted). The satisfaction and frustration of psychological needs in games will be reported using the Basic Needs in Games Scale, which includes 18 items (eg, ‘I could play the games in the way I wanted’) rated on a scale of 1=strongly disagree to 7=strongly agree.^79^ Immersion will be measured with the immersion subscale of the Player Experience Inventory, which includes three items (eg, ‘I was no longer aware of my surroundings while I was playing’) rated on a scale of 1=strongly disagree to 7=strongly agree.^80^ Prosocial behaviours in and out of games will be measured with three items adapted from Pronizius *et al*^81^ (eg, ‘Today, have you helped or supported a non-playable character in a video game?’; yes or no).

*Streaming*. Daily instances of streaming or watching game streams will be reported using two items similar to those for gaming. The satisfaction and frustration of psychological needs through streaming activities will be evaluated using a modified version of the Basic Needs in Games Scale.^79^ Immersion will be measured using a modified version of the immersion subscale of the Player Experience Inventory.^80^

### Patient and public involvement

This study is part of Game-in-Action, a larger project that aims to better understand the impacts of video games on the mental health of young people and to develop new intervention strategies. The project is based at the Ludic Mind Studio, a digital incubator led by the senior author, Dr Ferrari: https://www.mcgill.ca/ludicmind/. Based on the principles of integrated knowledge translation^82^ and the Strategy for Patient-Oriented Research framework,^83^ Game-in-Action engages youth, parents, researchers, clinicians and institutional-level and provincial-level decision-makers in designing the project and supporting its implementation. These frameworks ensure that the project activities and outcomes will ultimately achieve meaningful benefits for Quebec youth, for the adults close to them (eg, parents, educators, social/health providers), and for social and health services in the province. We established a multi-sectoral and multi-stakeholder Advisory Committee (AC) at the outset to provide oversight at all stages of the project. The AC includes members from the provincial Quebec health ministry, social and health services, the video game industry and youth of the Gaming Against Stigma Advisory Group (Youth Advisory). The Youth Advisory includes gamers aged between 18 and 35 years old, some with lived experience of mental illness.

The Youth Advisory and the Ludic Mind Studio research team meet every month to discuss their research activities and knowledge translation projects. The Youth Advisory was involved from the beginning of the present study (study design and application for funding) in April 2023. The rest of the AC was formed after obtaining funding in September 2023. Since then, we have been holding joint meetings with the AC approximately every 6 months, in addition to monthly meetings with the Youth Advisory. The AC and Youth Advisory contributed to the present study’s research questions by advocating for the inclusion of streaming-related measures based on their personal experiences of streaming as an important part of game culture. The AC also supported the foregrounding of social factors as part of the theoretical framework and research questions of the study, notably to acknowledge the role of games for coping with challenging life circumstances. The selection of measures was made by the research team based on these considerations and was presented to the AC, who approved it. The Youth Advisory supported recruitment by disseminating the study invitation in their networks. Members of the Youth Advisory tested the cross-sectional survey to confirm that the time for completion of the survey was acceptable, and some changes were made based on their feedback (eg, relabeling a ‘Clinical factors’ category, which includes psychotic-like experiences, as ‘Psychological factors’ instead). The Youth Advisory will be similarly involved in testing the longitudinal and mobile sensing measures, which are in the process of being implemented. After completion of the study, we will present our preliminary results to the AC to obtain their feedback and include their perspective in the interpretation and dissemination of the results.

### Analyses

We will conduct quantitative analyses in R (R Foundation for Statistical Computing). Characteristics of the analytic samples (cross-sectional and longitudinal) will be described to identify the potential underrepresentation of groups (eg, gender, ethnoracial, socioeconomic or rural vs urban groups) that may limit the generalisability of findings. Using lavaan,^84^ we will examine the factor structure and internal consistency of ad-hoc or modified questionnaires, and, for analyses of longitudinal changes over time, we will also test for longitudinal measurement invariance.^85^ Statistical significance will be defined as p<0.05 or 95% CIs not overlapping the null. We will apply correction for false discovery rate using the Benjamini and Hochberg^86^ method when multiple predictors are tested for a single hypothesised outcome (eg, there are two predictors of psychotic-like experiences for Objective 1: play time with family, friends or romantic partners, and the modified Fandom Functions Scale—Belonging). We will analyse cross-sectional outcomes using generalised linear models adjusted for relevant socio-demographic characteristics (eg, sex, educational attainment and occupational status). In these models, missing data will be handled by complete case analysis or multiple imputation. We will analyse longitudinal outcomes (eg, daily well-being) using generalised linear mixed models with observations nested in individuals. Missing data will be handled by maximum likelihood. To parse out within-person and between-person associations between longitudinal repeated measures (eg, the association between daily psychological need satisfaction and daily well-being), we will apply person-centring and mean-centring to the predictors; this approach accounts for time-invariant, unmeasured individual factors that could confound associations between the predictor and outcome.^55^ Mobile sensing data will be transformed into higher-level, validated features over epochs of 1 day for integration into generalised linear mixed models as exploratory predictors of game-related experiences and well-being; for example, geolocation data will be used to infer the daily amount of time spent at home.

Of note, because the digital phenotyping component will only start after completion of the cross-sectional sample, we anticipate that for some participants, the interval between the two data collections may be up to about 12 months. We will consider the impact of this delay on the interpretation of the eventual study findings. For example, the delay could attenuate the strength of the association between modified Fandom Functions Scale—Belonging (cross-sectional) and daily well-being (digital phenotyping) in Objective 1, given that the experience of belonging in game communities may change with time.

We will use reflexive thematic analysis for qualitative data.^87^ This approach aims to uncover shared patterns of meaning across participants’ responses through a reflexive, recursive process. The steps of reflexive thematic analysis include: (1) Familiarisation with the data, (2) Generating initial codes, (3) Searching for themes, (4) Reviewing potential themes and (5) Defining and naming the themes.^88^ Our analysis of the qualitative data will be primarily inductive to better understand the lived experiences of gamers. Finally, in collaboration with the AC, the quantitative and qualitative results will be integrated within a critical realist framework to support or revise the theoretical interpretations.^89^

## Ethics and dissemination

### Ethical considerations

The study will be conducted according to ethical principles stated in the Declaration of Helsinki and received ethical approval on 20 June 2024, from the Institutional Review Board of McGill University (reference number: 24-02-015). Consent forms will take into consideration the well-being, free will and respect of participants, including respect for privacy. For participants younger than age 18, caregiver consent is not always required in Canada. Here, for participants aged 16–18, we will not require consent from caregivers, based on the consideration that the risks of participation in the study are minimal, and that participants will be invited to provide data, which they may wish to keep private from their caregivers—such as information on gender identity, cannabis use, gaming habits and social relationships with peers.

### Safety considerations

Apps commonly used by adolescents and adults frequently collect and upload their geolocation data to the internet, often without users’ full awareness.^90^ For the current research, we are emphasising this aspect of the digital phenotyping component in the consent form to ensure participants’ full awareness and consent of the procedure. Data will be stored on secure servers located in Montreal, Canada, that are fully compliant with Quebec regulations on data privacy. Identifying information (name, email address, postal code) will be stored in a separate file linked to the rest of the data via an identification number. To protect the privacy of participants, geolocation data will be converted into non-identifying features: time spent at home (inferred based on the location most visited by the participant during the study period) and the amount of movement. Raw geolocation data will be deleted within 6 months of completion of data collection. Access to the processed, de-identified data will be limited to the research team, including students and collaborators. Data will be kept for 10 years before being deleted.

We also acknowledge that some questions may trigger discomfort or distress in participants. Participants will be informed that they can contact the research team at any point to receive assistance as needed, including immediate support and orientation to appropriate psychological or social services in the community.

### Dissemination plan

Results of this study will be disseminated through peer-reviewed publications and will be shared with gamers and people with lived experience of mental illness, in collaboration with the study’s Youth Advisory. The findings will inform the design of subsequent stages of the Game-in-Action project, which will include interventional research and will be described elsewhere. The results will also be presented to policymakers in the province of Quebec, Canada, via meetings organised by the Fonds de recherche du Quebec (the study sponsor) to inform policies regarding screen use among youth. Finally, the results will be presented to researchers and clinicians via workshops and talks in academic conferences, clinical rounds and other institutional events. While the present study aims to recruit participants from diverse backgrounds, it is likely that convenience sampling across groups will not be sufficient to uncover all potential group-based differences. A future direction for research will thus be to incorporate more targeted sampling strategies to better capture differences in gaming experiences related to sociocultural and personal circumstances, such as ethnoracial groups, LGBTQ+identities, mental health and disabilities.

## Supplementary material

10.1136/bmjopen-2025-103685 online supplemental file 1

## References

[1] ATN (2023). La famille numérique. NETendances.

[2] Pew Research Center (2024). Teens and video games today. https://www.pewresearch.org/internet/2024/05/09/teens-and-video-games-today/.

[3] ESAC (2022). Bringing canadians together through gaming: essential facts 2022. https://theesa.ca/resource/bringing-canadians-together-through-gaming-essential-facts-2022/.

[4] Clement J (2025). Video gaming worldwide. https://www.statista.com/topics/1680/gaming/.

[5] ESA (2024). Power of play – global report 2023. https://www.theesa.com/resources/power-of-play-global-report-2023/.

[6] ESAC (2020). What is a real canadian gamer? – real canadian gamers essential facts 2020. https://essentialfacts2020.ca/what-is-a-real-canadian-gamer/.

[7] Anderson CA, Bushman BJ (2001). Effects of violent video games on aggressive behavior, aggressive cognition, aggressive affect, physiological arousal, and prosocial behavior: a meta-analytic review of the scientific literature. Psychol Sci.

[8] Weis R, Cerankosky BC (2010). Effects of video-game ownership on young boys’ academic and behavioral functioning: a randomized, controlled study. Psychol Sci.

[9] Cain N, Gradisar M (2010). Electronic media use and sleep in school-aged children and adolescents: A review. Sleep Med.

[10] Sanders T, Noetel M, Parker P (2024). An umbrella review of the benefits and risks associated with youths’ interactions with electronic screens. Nat Hum Behav.

[11] Markey PM, Ferguson CJ, Hopkins LI (2020). Video Game Play: Myths and Benefits. Am J Play.

[12] World Health Organization (2019). International Classification of Diseases, eleventh revision (ICD-11). https://icd.who.int/browse11/l-m/en.

[13] Aarseth E, Bean AM, Boonen H (2017). Scholars’ open debate paper on the World Health Organization ICD-11 Gaming Disorder proposal. J Behav Addict.

[14] Billieux J, Flayelle M, Rumpf H-J (2019). High Involvement Versus Pathological Involvement in Video Games: a Crucial Distinction for Ensuring the Validity and Utility of Gaming Disorder. Curr Addict Rep.

[15] Király O, Koncz P, Griffiths MD (2023). Gaming disorder: A summary of its characteristics and aetiology. Compr Psychiatry.

[16] Huot-Lavoie M, Gabriel-Courval M, Béchard L (2023). Gaming Disorder and Psychotic Disorders: A Scoping Review. Psychopathology.

[17] Paquin V, Philippe FL, Shannon H (2024). Associations between digital media use and psychotic experiences in young adults of Quebec, Canada: a longitudinal study. Soc Psychiatry Psychiatr Epidemiol.

[18] Paquin V, Ferrari M, Rej S (2024). Trajectories of Adolescent Media Use and Their Associations With Psychotic Experiences. JAMA Psychiatry.

[19] Vuorre M, Ballou N, Hakman T (2024). Affective Uplift During Video Game Play: A Naturalistic Case Study. *ACM Games*.

[20] Egami H, Rahman MS, Yamamoto T (2024). Causal effect of video gaming on mental well-being in Japan 2020-2022. Nat Hum Behav.

[21] Giardina A, Schimmenti A, Starcevic V (2024). Problematic gaming, social withdrawal, and Escapism: The Compensatory-Dissociative Online Gaming (C-DOG) model. Comput Human Behav.

[22] Snodgrass JG (2023). The Avatar Faculty: Ecstatic Transformations in Religion and Video Games.

[23] Yee N (2006). The Demographics, Motivations, and Derived Experiences of Users of Massively Multi-User Online Graphical Environments. *Presence: Teleoperators and Virtual Environments*.

[24] Colder Carras M, Kalbarczyk A, Wells K (2018). Connection, meaning, and distraction: A qualitative study of video game play and mental health recovery in veterans treated for mental and/or behavioral health problems. Soc Sci Med.

[25] Ryan RM, Rigby CS, Przybylski A (2006). The Motivational Pull of Video Games: A Self-Determination Theory Approach. Motiv Emot.

[26] Granic I, Morita H, Scholten H (2020). Beyond Screen Time: Identity Development in the Digital Age. Psychol Inq.

[27] Pallavicini F, Ferrari A, Mantovani F (2018). Video Games for Well-Being: A Systematic Review on the Application of Computer Games for Cognitive and Emotional Training in the Adult Population. Front Psychol.

[28] Johnson MR (2019). Inclusion and exclusion in the digital economy: disability and mental health as a live streamer on Twitch.tv. Information, Communication & Society.

[29] Zsila Á, Shabahang R, Aruguete MS (2022). Toxic behaviors in online multiplayer games: Prevalence, perception, risk factors of victimization, and psychological consequences. Aggress Behav.

[30] Qian TY, Wang JJ, Zhang JJ (2022). Fulfilling the Basic Psychological Needs of Esports Fans: A Self-Determination Theory Approach. *Communication & Sport*.

[31] Paquin V, Ferrari M, Sekhon H (2023). Time to Think “Meta”: A Critical Viewpoint on the Risks and Benefits of Virtual Worlds for Mental Health. JMIR Serious Games.

[32] Ballou N, Hakman T, Vuorre M (2024). How do video games affect mental health? a narrative review of 13 proposed mechanisms. *PsyArXiv*.

[33] Gómez-Carrillo A, Kirmayer LJ (2023). A cultural-ecosocial systems view for psychiatry. Front Psychiatry.

[34] Kirmayer LJ (2025). The place of the social in psychiatry: from structural determinants to the ecology of mind. *Soc Psychiatry Psychiatr Epidemiol*.

[35] Gómez-Carrillo A, Paquin V, Dumas G (2023). Restoring the missing person to personalized medicine and precision psychiatry. Front Neurosci.

[36] Gómez-Carrillo A, Kirmayer LJ, Aggarwal NK (2023). Integrating neuroscience in psychiatry: a cultural-ecosocial systemic approach. Lancet Psychiatry.

[37] Johannes N, Vuorre M, Przybylski AK (2021). Video game play is positively correlated with well-being. R Soc Open Sci.

[38] Richard J, Temcheff CE, Derevensky JL (2020). Gaming Disorder Across the Lifespan: a Scoping Review of Longitudinal Studies. Curr Addict Rep.

[39] Bodenstein KC (2023). Biomarkers in Neuropsychiatry: A Primer.

[40] De Angel V, Lewis S, White K (2022). Digital health tools for the passive monitoring of depression: a systematic review of methods. *NPJ Digit Med*.

[41] Jennett C, Cox AL, Cairns P (2008). Measuring and defining the experience of immersion in games. Int J Hum Comput Stud.

[42] Vella K, Johnson D, Hides L Positively playful: when videogames lead to player wellbeing.

[43] Wang H-Y, Cheng C (2022). The Associations Between Gaming Motivation and Internet Gaming Disorder: Systematic Review and Meta-analysis. JMIR Ment Health.

[44] Snodgrass JG, Lacy MG (2011). Cultural Consonance and Mental Wellness in the World of Warcraft: Online Games as Cognitive Technologies of ‘Absorption-Immersion. Cogn Technol.

[45] Nagata JM, Singh G, Sajjad OM (2022). Social epidemiology of early adolescent problematic screen use in the United States. Pediatr Res.

[46] Peat G, Rodriguez A, Smith J (2023). “It is easier to not allow them to see your disability straight away, to see you as a person”: An Interpretative Phenomenological Analysis of video gaming from the perspectives of men with Duchenne Muscular Dystrophy. Palliat Med.

[47] Hygen BW, Wendelborg C, Solstad BE (2024). Gaming motivation and well-being among Norwegian adult gamers: the role of gender and disability. *Front Med Technol*.

[48] Cote AC (2020). Gaming Sexism: Gender and Identity in the Era of Casual Video Games.

[49] Gray KL (2020). Intersectional Tech: Black Users in Digital Gaming.

[50] Snodgrass JG, Lacy MG, Francois Dengah HJ (2011). Enhancing one life rather than living two: Playing MMOs with offline friends. Comput Human Behav.

[51] Taylor K (2024). The social diagnoses of digital addictions: Technophobic ambivalences, the limits of the natural and imperatives of self‐governance in the information age. Sociology Health & Illness.

[52] Rautalahti H (2019). “How video games changed my life”: life-changing testimonies and the last of us. Gamevironments.

[53] Staines L, Hoey J, Cannon M (2024). Identifying and Using Psychotic-Like Experiences in Clinical Practice and Public Policy. Biological Psychiatry: Cognitive Neuroscience and Neuroimaging.

[54] Vandenbroucke JP, Elm E von, Altman DG (2007). Strengthening the Reporting of Observational Studies in Epidemiology (STROBE): Explanation and Elaboration. Ann Intern Med.

[55] Myin-Germeys I, Kuppens P (2022). The Open Handbook of Experience Sampling Methodology: A Step-by-Step Guide to Designing, Conducting, and Analyzing ESM Studies.

[56] Barnett I, Torous J, Reeder HT (2020). Determining sample size and length of follow-up for smartphone-based digital phenotyping studies. J Am Med Inform Assoc.

[57] Currey D, Torous J (2022). Digital phenotyping correlations in larger mental health samples: analysis and replication. BJPsych Open.

[58] Allen JJ (2020). Gaming as psychologically nutritious: does need satisfaction in video games contribute to daily well-being beyond need satisfaction in the real world.

[59] Chadborn D, Edwards P, Reysen S (2017). Displaying Fan Identity to Make Friends. Intensities J Cult Media.

[60] Reinecke L (2009). Games and Recovery. J Media Psychol.

[61] Burke B, Lucier-Greer M (2021). Comparing video game engagement measures as related to individual and relational well-being in a community sample of adult gamers. *Computers in Human Behavior Reports*.

[62] Zabriskie RB, McCormick BP (2001). The Influences of Family Leisure Patterns on Perceptions of Family Functioning. Fam Relat.

[63] Sherry JL, Lucas K, Greenberg BS (2006). Playing Video Games: Motives, Responses, and Consequences.

[64] Pontes HM, Schivinski B, Sindermann C (2021). Measurement and Conceptualization of Gaming Disorder According to the World Health Organization Framework: the Development of the Gaming Disorder Test. Int J Ment Health Addiction.

[65] Olweus D, Limber SP, Breivik K (2019). Addressing Specific Forms of Bullying: A Large-Scale Evaluation of the Olweus Bullying Prevention Program. *Int Journal of Bullying Prevention*.

[66] Jamieson GA (2005). The Modified Tellegen Absorption Scale: A clearer window on the structure and meaning of absorption. Aust J Clin Exp Hypn.

[67] Parsons TD, Barnett M, Melugin PR (2015). Assessment of Personality and Absorption for Mediated Environments in a College Sample. Cyberpsychol Behav Soc Netw.

[68] Lifshitz M, van Elk M, Luhrmann TM (2019). Absorption and spiritual experience: A review of evidence and potential mechanisms. Conscious Cogn.

[69] Rammstedt B, John OP (2007). Measuring personality in one minute or less: A 10-item short version of the Big Five Inventory in English and German. J Res Pers.

[70] Courtois R, Petot J-M, Plaisant O (2020). Validation française du Big Five Inventory à 10 items (BFI-10). L’Encéphale.

[71] Capra C, Kavanagh DJ, Hides L (2017). Current CAPE-15: a measure of recent psychotic-like experiences and associated distress. Early Interv Psychiatry.

[72] Brenner K, Schmitz N, Pawliuk N (2007). Validation of the English and French versions of the Community Assessment of Psychic Experiences (CAPE) with a Montreal community sample. Schizophr Res.

[73] Kocalevent R-D, Berg L, Beutel ME (2018). Social support in the general population: standardization of the Oslo social support scale (OSSS-3). BMC Psychol.

[74] Landry M, Tremblay J, Guyon L (2004). La Grille de dépistage de la consommation problématique d’alcool et de drogues chez les adolescents et les adolescentes (DEP-ADO) : développement et qualités psychométriques. *dss*.

[75] Fried EI, Proppert RKK, Rieble CL (2023). Building an Early Warning System for Depression: Rationale, Objectives, and Methods of the WARN-D Study. Clin Psychol Eur.

[76] Roenneberg T, Wirz-Justice A, Merrow M (2003). Life between clocks: daily temporal patterns of human chronotypes. J Biol Rhythms.

[77] Eisele G, Lafit G, Vachon H (2021). Affective structure, measurement invariance, and reliability across different experience sampling protocols. J Res Pers.

[78] Gentile DA, Lynch PJ, Linder JR (2004). The effects of violent video game habits on adolescent hostility, aggressive behaviors, and school performance. J Adolesc.

[79] Ballou N, Denisova A, Ryan R (2024). The Basic Needs in Games Scale (BANGS): A new tool for investigating positive and negative video game experiences. Int J Hum Comput Stud.

[80] Abeele VV, Spiel K, Nacke L (2020). Development and validation of the player experience inventory: A scale to measure player experiences at the level of functional and psychosocial consequences. Int J Hum Comput Stud.

[81] Pronizius E, Forbes PAG, Feneberg AC (2024). Everyday helping is associated with enhanced mood but greater stress when it is more effortful. Sci Rep.

[82] Andrews D, Fong G, Hackam D (2012). Guide to Knowledge Translation Planning at CIHR: Integrated and End-of-Grant Approaches. Canadian Institutes of Health Research.

[83] Tricco AC, Zarin W, Clement F (2022). Introducing the Strategy for Patient Oriented Research (SPOR) Evidence Alliance: a partnership between researchers, patients and health system decision-makers to support rapid-learning and responsive health systems in Canada and beyond. Facets (Ott).

[84] Rosseel Y, Jorgensen TD, Rockwood N (2022). Lavaan: latent variable analysis.

[85] McNeish D, Mackinnon DP, Marsch LA (2021). Measurement in Intensive Longitudinal Data. *Structural Equation Modeling: A Multidisciplinary Journal*.

[86] Benjamini Y, Hochberg Y (1995). Controlling the False Discovery Rate: A Practical and Powerful Approach to Multiple Testing. Journal of the Royal Statistical Society Series B.

[87] Braun V, Clarke V (2019). Reflecting on reflexive thematic analysis. *Qualitative Research in Sport, Exercise and Health*.

[88] Braun V, Clarke V, Cooper H, Camic PM, Long DL APA Handbook of Research Methods in Psychology, Vol 2: Research Designs: Quantitative, Qualitative, Neuropsychological, and Biological.

[89] Fletcher AJ (2017). Applying critical realism in qualitative research: methodology meets method. Int J Soc Res Methodol.

[90] Hong JI Designing for Privacy in Mobile Sensing Systems. Mobile Sensing in Psychology: Methods and Applications.

