# Supplementary material for: Understanding the game experiences and mental health of youth: protocol for the Game-in-Action Quebec cohort study
Source: BMJ Open. 2025 Sep 1;15(9):e103685. doi: 10.1136/bmjopen-2025-103685 (PMC12406825; doi:10.1136/bmjopen-2025-103685)
Supplement: online supplemental file 1 [file bmjopen-15-9-s001.docx]

**Game-in-Action Quebec Cohort Study – Questionnaires**

**Cross-sectional survey (n=1000)**

| **Questions** | **Sources** |
| --- | --- |
| ***Eligibility questions*** | |
| 1. What is your age?   [Dropdown] 15 years or younger  16  17  18  19  20  21  22  23  24  25  26 years or older   1. What is your year of birth?   [Textbox]   1. Do you currently live in the Province of Québec, Canada?    1. [Radio buttons]       1. Yes       2. No 2. Many people regularly play video games on computers (e.g. Minecraft), smartphones (e.g. Candy Crush), and gaming consoles (e.g. New Super Mario Bros.).   In a typical week, how much time do you usually spend playing video games?   - 1. [Radio buttons]      1. None      2. Less than 1 hour per week      3. From 1 to 2 hours per week      4. From 3 to 5 hours per week      5. From 6 to 10 hours per week      6. From 11 to 14 hours per week      7. From 15 to 20 hours per week      8. More than 20 hours per week | Questions 1-3  Ad hoc  Question 4:  Preface (“Many people regularly play video games…”) adapted from: Przybylski, A. K., & Weinstein, N. (2019). Investigating the Motivational and Psychosocial Dynamics of Dysregulated Gaming: Evidence from a Preregistered Cohort Study. *Clinical Psychological Science*, *7*(6), 1257-1265.  Response options adapted from the Quebec Longitudinal Study of Child Development: <https://www.jesuisjeserai.stat.gouv.qc.ca/>. |
| ***Sociodemographic variables*** | |
| 1. What was your sex assigned at birth?    1. [Radio buttons]       1. Female       2. Male       3. Intersex       4. I prefer not to say 2. Which term(s) best describe your current gender identity? [Select all the option(s) that apply to you.]    1. [Radio buttons]       1. Woman       2. Man       3. Non-binary       4. Two-spirit       5. Other (Please Specify) [Textbox]       6. I prefer not to say 3. Select the population group(s) you identify with.    1. [Radio buttons; Checkboxes]       1. Arab or Middle Eastern       2. Asian       3. Black or African       4. Hispanic or Latinx       5. Indigenous       6. White       7. Other (Please Specify) [Textbox] 4. What is your highest level of education completed?    1. [Dropdown menu]       1. None       2. Secondary school certificate of the equivalent (e.g., SSD Secondary School Diploma)       3. Vocational or trade school certificate or diploma (e.g., DVS – Diploma of Vocational Studies)       4. College certificate or diploma (non-university certificate or diploma obtained from a CEGEP, community college, technical institute, etc.)       5. University certificate or diploma below bachelor level       6. Bachelor’s degree (e.g., B.A., B.Sc., LL.B.)       7. University certificate or diploma above bachelor level       8. Master’s degree (e.g., M.A., M.Sc., M.Ed.)       9. Degree in medicine, dentistry, veterinary medicine or optometry (M.D., D.D.S., D.M.D., D.M.V., O.D.)       10. Earned doctorate (e.g., Ph. D., D.Sc., D.Ed.)       11. Other (specify): _____ 5. Are you currently a student? 6. [Radio buttons]    - 1. Yes, full time      2. Yes, part time      3. No 7. Are you currently employed? 8. [Radio buttons]    - 1. Yes, full time      2. Yes, part time      3. No 9. What is your postal code? 10. [Textbox] 11. [Subtext:] This information is optional and will be stored separately from your other responses. It will be used to evaluate characteristics of your area of residence such as population density. 12. [subtext:] format: A1D 7T5 13. Do you identify as someone with a disability? 14. Yes 15. No 16. I prefer not to answer 17. Currently or in the past, have you had any mental health condition, whether diagnosed or not by a health professional? 18. [Radio buttons]     - 1. Yes       2. No       3. Prefer not to answer 19. [Conditional, if previous answer was “Yes”] What were those mental health conditions? 20. [Textbox] 21. Currently or in the past, have you sought out help/services for mental health? 22. [Radio buttons]     - 1. Yes       2. No       3. Prefer not to answer | Questions 5-6, sex and gender:  Adapted from list of items in Table 6A-1, in:  Becker, T., Chin, M., Bates, N., & National Academies of Sciences, Engineering, and Medicine. (2022). Measuring Sex and Gender Identity. In Measuring Sex, Gender Identity, and Sexual Orientation. *National Academies Press* (US).  Question 7, ethnoracial categories:  Adapted from:  Anglin, D. M., & Lui, F. (2023). Racial microaggressions and major discriminatory events explain ethnoracial differences in psychotic experiences. *Schizophrenia research*, *253*, 5-13.  Paquin, V., Philippe, F. L., Shannon, H., Guimond, S., Ouellet-Morin, I., & Geoffroy, M. C. (2024). Associations between digital media use and psychotic experiences in young adults of Quebec, Canada: a longitudinal study. *Social Psychiatry and Psychiatric Epidemiology*, *59*(1), 65-75.  Question 8, highest level of education:  Paquin, V., Philippe, F. L., Shannon, H., Guimond, S., Ouellet-Morin, I., & Geoffroy, M. C. (2024). Associations between digital media use and psychotic experiences in young adults of Quebec, Canada: a longitudinal study. *Social Psychiatry and Psychiatric Epidemiology*, *59*(1), 65-75.  Questions 9-10, current student and employment status:  Paquin, V., Philippe, F. L., Shannon, H., Guimond, S., Ouellet-Morin, I., & Geoffroy, M. C. (2024). Associations between digital media use and psychotic experiences in young adults of Quebec, Canada: a longitudinal study. *Social Psychiatry and Psychiatric Epidemiology*, *59*(1), 65-75.  Questions 11-12:  Ad hoc  Question 13-15, mental health problems:  Paquin, V., Philippe, F. L., Shannon, H., Guimond, S., Ouellet-Morin, I., & Geoffroy, M. C. (2024). Associations between digital media use and psychotic experiences in young adults of Quebec, Canada: a longitudinal study. *Social Psychiatry and Psychiatric Epidemiology*, *59*(1), 65-75. |
| ***Video gaming habits*** | |
| 1. Do you consider yourself a gamer?    - 1. Yes      2. No 2. At what age did you start playing video games regularly?   [Textbox] years  How often do you play the following genres of video games?  [Radio buttons] *Scale: 1 = Never, 2 = Rarely, 3 = Sometimes, 4 = Often, 5 = Very Often*   1. action/adventure 2. sports 3. role-playing 4. strategy 5. simulation 6. puzzle 7. shooter 8. racing 9. fighting 10. massively-multiplayer online 11. platformer 12. music & dance 13. Other (Please specify) [Textbox]   People may like video games for various reasons. Think about the times you feel the most connected to and/or engaged with video games or the game community and rate the following items.  My involvement with video games and the game community…  [Radio buttons] *Scale: 1 = Not at all true, 2, 3, 4, 5 = Very true*   1. Provides me with an opportunity to grow and discover more about aspects of myself. 2. Provides me with answers/information/guidance I need to face situations in my life. 3. Provides me with an experience of captivating beauty and inspiration. 4. Provides me with a sense of fulfillment as I feel like something is missing when I am not involved. 5. Provides me with an opportunity to express myself and my uniqueness. 6. Provides me with an outlet for my creativity. 7. Provides me with a focus or sense of purpose. 8. Provides me with a break from life's stresses. 9. Provides me with an engaging and entertaining experience. 10. Provides me with a good laugh and/or cry. 11. Provides me with a chance/way to retreat from difficult life situations. 12. Provides me an opportunity to share with my family and bring us closer. 13. Provides me with an activity to share with my existing friends/ ways to stay connected. 14. Provides me with a chance to expand my circle of friends. 15. If you can, please elaborate on your reason(s) for gaming.   [Textbox]  Please rate the following items regarding your experience when playing video games.  [Radio buttons] *1 = Does not apply at all, 5 = Does fully apply*   1. When I play video or computer games I forget about work. 2. When I play video or computer games I use the time to relax. 3. When I play video or computer games I do things that challenge me. 4. When I play video or computer games I feel like I can decide for myself what to do.   [Radio buttons] *0 = No participation, 1 = At least annually, 2 = At least monthly, 3 = At least weekly, 4 = At least daily*   1. How frequently do you engage in game play with family members, friends, or romantic partners (whether physically present or through Internet-mediated communication channels)? 2. How frequently did you engage in game play with family members, friends, or romantic partners over the past year? 3. Indicate the general length (in hours) of each play session when playing with family members, friends, or romantic partners.   [Textbox]  In the following you will find a number of statements concerning activities and experiences you can engage with during your leisure time. Please rate for each statement how much it applies to you personally with regard to playing video and computer games.  While playing video and computer games…  [Radio buttons] *Scale: 1 = Strongly Disagree, 5 = Strongly Agree*   1. I like to play to prove to my friends that I am the best. 2. When I lose to someone, I immediately want to play again in an attempt to beat him/her/them. 3. It is important to me to be the fastest and most skilled person playing the game. 4. I get upset when I lose to my friends. 5. I feel proud when I master an aspect of a game. 6. I find it very rewarding to get to the next level. 7. I play until I complete a level or win a game. 8. I enjoy finding new and creative ways to work through video games. 9. My friends and I use video games as a reason to get together. 10. Often, a group of friends and I will spend time playing video games. 11. I play video games when I have other things to do. 12. I play video games instead of other things I should be doing. 13. I play video games because they let me do things I can’t do in real life. 14. Video games allow me to pretend I am someone/somewhere else. 15. I like to do something that I could not normally do in real life through a video game. 16. I enjoy the excitement of assuming an alter ego in a game. 17. I find that playing video games raises my level of adrenaline. 18. Video games keep me on the edge of my seat. 19. I play video games because they stimulate my emotions. 20. I play video games because they excite me. 21. What do you like about playing video games?   [Textbox]   1. If you are able, please describe a time where playing video games helped your mental health.   [Textbox]   1. If you are able, please describe a time where playing video games worsened your mental health.   [Textbox]   1. What was your favourite video game growing up?   [Textbox]   1. What is your favourite video game at the moment?   [Textbox]   1. What are your favourite 3 video games ever? Indicate in percentages how much you played each of the three games (e.g., Call of Duty 45%, Super Mario 50%, Minecraft 5%, )   [Textbox]   1. If you could be any video game character, who would you be and why?   [Textbox]   1. Do you feel gaming has an impact on your daily life and well-being?   [Textbox]  Please indicate how often the following issues occurred on average over the past twelve months until today.  [Radio buttons] *Scale: 1 = Never, 2 = Rarely, 3 = Sometimes, 4 = Often, 5 = Very Often*   1. I have difficulties controlling my gaming activity. 2. I have given increasing priority to gaming over other life interests and daily activities. 3. I have continued gaming despite the occurrence of negative consequences. 4. I have experienced significant problems in life (e.g., personal, family, social, education, occupational) due to the severity of my gaming behaviour.   Here are some questions about being bullied by other gamers, either in real life or in a virtual space. First, we define or explain the word bullying. We say someone is being bullied when another person:  • says mean and hurtful things or make fun of him/her/them or calls him/her/them mean and hurtful names;  • completely ignores or excludes him/her/them from their group of friends or leaves him/her/them out of things on purpose;  • hits, kicks, pushes, shoves around, or locks him/her/them inside a room;  • tells lies or spreads false rumors about him/her/them  • sends mean notes and tries to make other people dislike him/her/them;  • and says or does hurtful things like that.   1. How often have you been bullied while gaming in the past couple of months? 2. It hasn’t happened to me in the past couple months 3. Only once or twice in the past couple months 4. 2 or 3 times a month 5. About once a week 6. Several times a week 7. How often were you called mean names, made fun of, or teased in a hurtful way while gaming during the past couple of months? 8. It hasn’t happened to me in the past couple months 9. Only once or twice in the past couple months 10. 2 or 3 times a month 11. About once a week 12. Several times a week 13. How often have you taken part in bullying another gamer(s) while gaming during the past couple of months? 14. I haven’t bullied another gamer(s) in the past couple months 15. Only once or twice in the past couple months 16. 2 or 3 times a month 17. About once a week 18. Several times a week | Question 18-30, preferred game genres:  Adapted from:  Allen, J. J. (2020). Gaming as psychologically nutritious: Does need satisfaction in video games contribute to daily well-being beyond need satisfaction in the real world? (Doctor of Philosophy, Iowa State University). Iowa State University. <https://doi.org/10.31274/etd-20200902-2>  *Modifications:*   - Allen provides 3 examples per genre of recent games. Not included here. - The response scale was adapted for consistency with the subsequent gaming disorder test.   Questions 31-44, Fandom Functions scale:  Chadborn, D., Edwards, P., & Reysen, S. (2017). Displaying fan identity to make friends. *Intensities:* *The Journal of Cult Media, 9,* 87-97.  *Modifications:*   - The original prompt was shortened and adapted for video games.   Questions 45:  Ad hoc  Questions 46-49, Psychological Recovery Scale:  Adapted from:  Reinecke, L. (2009). Games and recovery: The use of video and computer games to recuperate from stress and strain. *Journal of media psychology*, *21*(3), 126-142.  Questions 50-52, Family Leisure Activity Profile:  Zabriskie, R. B., & McCormick, B. P. (2001). The influences of family leisure patterns on perceptions of family functioning. *Family Relations*, *50*(3), 281-289.  Adaptation in:  Burke, B., & Lucier-Greer, M. (2021). Comparing video game engagement measures as related to individual and relational well-being in a community sample of adult gamers. *Computers in Human Behavior Reports*, *4*, 100136.  Questions 53-72, Video Game Uses and Gratifications Instrument:  Sherry, J. L., Lucas, K., Greenberg, B. S., & Lachlan, K. (2006). Video Game Uses and Gratifications as Predicators of Use and Game Preference. In *Playing video games: Motives, responses, and consequences* (pp. 213–224). Lawrence Erlbaum Associates Publishers.    Questions 73-80:  Ad hoc  Questions 81-84, Gaming Disorder Test:  Pontes, H. M., Schivinski, B., Sindermann, C., Li, M., Becker, B., Zhou, M., & Montag, C. (2021). Measurement and conceptualization of Gaming Disorder according to the World Health Organization framework: The development of the Gaming Disorder Test. *International Journal of Mental Health and Addiction*, *19*, 508-528.  Questions 85-87, bullying:  Adapted from:  Olweus, D. (2007). Olweus bullying questionnaire: Standard school report. |
| ***Streaming habits*** | |
| 1. Do you consider yourself a streamer and/or someone who watches video game streams?    - 1. Yes      2. No   If “No”: Skip to next section.   1. Do you consider yourself    1. a streamer    2. someone who watches video game streams    3. both 2. In a typical week, how much time do you usually spend watching other people play video games (e.g. on Twitch or Youtube)?    1. [Radio buttons]       1. None       2. Less than 1 hour per week       3. From 1 to 2 hours per week       4. From 3 to 5 hours per week       5. From 6 to 10 hours per week       6. From 11 to 14 hours per week       7. From 15 to 20 hours per week       8. More than 20 hours per week 3. In a typical week, how much time do you usually spend streaming video games (e.g. on Twitch or Youtube)?    1. [Radio buttons]       1. None       2. Less than 1 hour per week       3. From 1 to 2 hours per week       4. From 3 to 5 hours per week       5. From 6 to 10 hours per week       6. From 11 to 14 hours per week       7. From 15 to 20 hours per week       8. More than 20 hours per week 4. At what age did you start watching video game streams regularly?   [Textbox] years   1. At what age did you start streaming regularly?   [Textbox] years   1. How often do you interact with video game streaming in the following roles?   [Radio buttons] *Scale: 1 = Never, 2 = Rarely, 3 = Sometimes, 4 = Often, 5 = Very Often*  Stream viewer  Stream moderator  Stream performer/player  How often do you watch streams or stream the following genres of video games?  [Radio buttons] *Scale: 1 = Never, 2 = Rarely, 3 = Sometimes, 4 = Often, 5 = Very Often*   1. Action/adventure 2. Sports 3. role-playing 4. Strategy 5. Simulation 6. Puzzle 7. Shooter 8. Racing 9. Fighting 10. massively-multiplayer online 11. Platformer 12. music & dance 13. Other (Please specify) [Textbox]   How often do you use the following streaming platforms?  [Radio buttons] *Scale: 1 = Never, 2 = Rarely, 3 = Sometimes, 4 = Often, 5 = Very Often*   1. Twitch 2. Kick 3. Caffeine 4. Facebook Gaming 5. Mobcrush 6. Owncast 7. Youtube 8. Other (Please specify) [Textbox]   People may like streaming or watching video game streams for various reasons. Think about the times you feel the most connected to and/or engaged with video game streaming or the streaming community and rate the following items.  My involvement with streaming or watching video game streams and the streaming community…  [Radio buttons] *Scale: 1 = Not at all true, 2, 3, 4, 5 = Very true*   1. Provides me with an opportunity to grow and discover more about aspects of myself. 2. Provides me with answers/information/guidance I need to face situations in my life. 3. Provides me with an experience of captivating beauty and inspiration. 4. Provides me with a sense of fulfillment as I feel like something is missing when I am not involved. 5. Provides me with an opportunity to express myself and my uniqueness. 6. Provides me with an outlet for my creativity. 7. Provides me with a focus or sense of purpose. 8. Provides me with a break from life's stresses. 9. Provides me with an engaging and entertaining experience. 10. Provides me with a good laugh and/or cry. 11. Provides me with a chance/way to retreat from difficult life situations. 12. Provides me an opportunity to share with my family and bring us closer. 13. Provides me with an activity to share with my existing friends/ ways to stay connected. 14. Provides me with a chance to expand my circle of friends. 15. If you can, please elaborate on your reason(s) for using streaming platforms.   [Textbox]  Please rate the following items regarding your experience when streaming and/or watching video game streams.  [Radio buttons] *1 = Does not apply at all, 5 = Does fully apply*   1. When I stream or watch video or computer games I forget about work. 2. When I stream or watch video or computer games I use the time to relax. 3. When I stream or watch video or computer games I do things that challenge me. 4. When I stream or watch video or computer games I feel like I can decide for myself what to do.   [Radio buttons] *0 = No participation, 1 = At least annually, 2 = At least monthly, 3 = At least weekly, 4 = At least daily*   1. How frequently do you stream or watch video game streams with family members, friends, or romantic partners (whether physically present or through Internet-mediated communication channels)? 2. How frequently did you stream or watch video game streams with family members, friends, or romantic partners over the past year? 3. Indicate the general length (in hours) of each play session when playing with family members, friends, or romantic partners.   [Textbox]  In the following you will find a number of statements concerning activities and experiences you can engage with during your leisure time. Please rate for each statement how much it applies to you personally, either as the streamer yourself or watching someone else stream, with regard to streaming or watching video and computer game streams.  While streaming or watching computer game streams…  [Radio buttons] *Scale: 1 = Strongly Disagree, 5 = Strongly Agree*   1. I like to stream and/or watch video game streams to prove to my friends that the streamer (myself or someone else) is the best. 2. When the streamer (myself or someone else) loses to someone, I immediately want them to play again in an attempt to beat their opponent. 3. It is important to me that the streamer (myself or someone else) be the fastest and most skilled person playing the game. 4. I get upset when the streamer (myself or someone else) loses to other players. 5. I feel proud when the streamer (myself or someone else) masters an aspect of a game. 6. I find it very rewarding to see the streamer (myself or someone else) get to the next level. 7. I stream and/or watch video game streams until the streamer (myself or someone else) completes a level or wins a game. 8. I enjoy finding new and creative ways to work through streaming and/or watching video game streams. 9. My friends and I use streaming and/or watching video game streams as a reason to get together. 10. Often, a group of friends and I will spend time streaming or watching video game streams. 11. I stream and/or watch video game streams when I have other things to do. 12. I stream and/or watch video game streams instead of other things I should be doing. 13. I stream and/or watch video game streams because they let me do things I can’t do in real life. 14. Stream or watching video game streams allow me to pretend I am someone/somewhere else. 15. I like to do something that I could not normally do in real life through streaming and/or watching video game streams. 16. I enjoy the excitement of assuming an alter ego while streaming and/or watching video game streams. 17. I find that streaming and/or watching video game streams raises my level of adrenaline. 18. Video game streams keep me on the edge of my seat. 19. I stream and/or watch video game streams because they stimulate my emotions. 20. I stream and/or watch video game streams because they excite me. 21. What do you like about streaming or watching video game streams?   [Textbox]   1. If you are able, please describe a time where streaming or watching video game streams helped your mental health.   [Textbox]   1. If you are able, please describe a time where streaming or watching video game streams deteriorated your mental health.   [Textbox]   1. What do you like about streaming or watching video game streams?   [Textbox]   1. Who was your favourite streamer growing up?   [Textbox]   1. What is your favourite streamer at the moment?   [Textbox]   1. If you could be any streamer, who would you be and why?   [Textbox]   1. Do you feel streaming or watching video game streams has an impact on your daily life and well-being?   [Textbox]  Please indicate how often the following issues occurred on average over the past twelve months until today.  [Radio buttons] *Scale: 1 = Never, 2 = Rarely, 3 = Sometimes, 4 = Often, 5 = Very Often*   1. I have difficulties controlling my streaming activity (includes watching video game streams). 2. I have given increasing priority to streaming or watching video game streams over other life interests and daily activities. 3. I have continued streaming or watching video game streams despite the occurrence of negative consequences. 4. I have experienced significant problems in life (e.g., personal, family, social, education, occupational) due to the severity of my streaming behaviour (includes watching video game streams).   Here are some questions about being bullied by other gamers/streamers/stream viewers, either in real life or in a virtual space. First, we define or explain the word bullying. We say someone is being bullied when another person:  • says mean and hurtful things or make fun of him/her/them or calls him/her/them mean and hurtful names;  • completely ignores or excludes him/her/them from their group of friends or leaves him/her/them out of things on purpose;  • hits, kicks, pushes, shoves around, or locks him/her/them inside a room;  • tells lies or spreads false rumors about him/her/them  • sends mean notes and tries to make other people dislike him/her/them;  • and says or does hurtful things like that.     1. How often have you been bullied while streaming or watching video game streams in the past couple of months? 2. It hasn’t happened to me in the past couple months 3. Only once or twice in the past months 4. 2 or 3 times a month 5. About once a week 6. Several times a week 7. How often were you called mean names, made fun of, or teased in a hurtful way while streaming or watching video game streams during the past couple of months? 8. It hasn’t happened to me in the past couple months 9. Only once or twice in the past months 10. 2 or 3 times a month 11. About once a week 12. Several times a week 13. How often have you taken part in bullying another streamer(s) or stream viewer(s) while streaming or watching video game streams during the past couple of months? 14. I haven’t bullied another streamer(s) or stream viewer(s) in the past couple months 15. Only once or twice in the past months 16. 2 or 3 times a month 17. About once a week 18. Several times a week | Sources are the same as those in the “Video gaming habits” section. The questionnaires were adapted to focus on streaming. |
| ***Personality*** | |
| Please fill in the following questionnaire by selecting the number that corresponds to how frequently each of the following statements is true of your experience. It is imperative that you answer these questions as honestly as possible and in accordance with your experiences. *Scale: 1 = Never, 5 = Always*   1. I feel and experience things as I did when I was a child. 2. I can be greatly moved by eloquent or poetic language. 3. While watching a movie, a TV show, or a play, I become so involved that I forget about myself and experience the story as if it were real and as if I were taking part in it. 4. If I stare at a picture and then look away from it, I can see an image of the picture, almost as if I were still looking at it. 5. I feel as if my mind could envelop the whole earth. 6. I like to watch cloud shapes in the sky. 7. If I wish, I can imagine (or daydream) some things so vividly that they hold my attention as a good movie or a story does. 8. I think I really know what some people mean when they talk about mystical experiences. 9. I can step outside my usual self and experience an entirely different state of being. 10. Textures – such as wool, sand, wood – remind me of colours. 11. I experience things as if they were doubly real. 12. When I listen to music, I can get so caught up in it that I don’t notice anything else. 13. If I wish, I can imagine that my whole body is so heavy that I could not move it if I wanted to. 14. I can somehow sense the presence of another person before I actually see or hear him/her. 15. The crackle and flames of a woodfire stimulates my imagination. 16. It is possible for me to be completely immersed in nature or art and to feel as if my whole state of consciousness has somehow been temporarily altered. 17. Different colours have distinctive special meanings for me. 18. I am able to wander off into my own thought while doing a routine task and actually forget that I am doing the task, and then find a few minutes later that I have completed it. 19. I can recollect certain past experiences in my life with such clarity and vividness that it is like living them again or almost so. 20. Things that might seem meaningless to others often make sense to me. 21. While acting in a play, I think I would really feel the emotions of the character and become her/him for the time being, forgetting both myself and the audience. 22. My thoughts do not occur as words but as visual images. 23. I take delight in small things (like the five pointed star shape that appears when you cut an apple across the core or the colours in soap bubbles). 24. When listening to organ music or other powerful music, I feel as if I am being lifted into the air. 25. I can change noise into music by the way I listen to it. 26. Some of my most vivid memories are called up by scents and smells. 27. Certain pieces of music remind me of pictures or patterns of colour. 28. I know what someone is going to say before he or she says it. 29. I have physical memories; for example, after I have been swimming I may still feel as if I am in the water. 30. The sound of a voice can be so fascinating to me that I can just go on listening to it. 31. I somehow feel the presence of someone who is not physically there. 32. Thoughts and images come to me without the slightest effort on my part. 33. I find that different odours have different colours. 34. I can be deeply moved by a sunset.   How well do the following statements describe your personality?  I see myself as someone who…  [Radio buttons] *Scale: 1 = Disagree strongly, 2 = Disagree a little, 3 = Neither Agree nor disagree, 4 = Agree a little, 5 = Agree strongly*   1. ... is reserved 2. ... is generally trusting 3. ... does a thorough job 4. ... is relaxed, handles stress well 5. ... has an active imagination 6. ... is outgoing, sociable 7. ... tends to find fault with others 8. ... tends to be lazy 9. ... gets nervous easily 10. ... has few artistic interests | Questions 173-206, Modified Tellegen Absorption Scale:  Jamieson, G. A. (2005). The modified tellegen absorption scale: A clearer window on the structure and meaning of absorption. Australian Journal of Clinical & Experimental Hypnosis, *33*(2), 119–139.  *French version:* Kyle Greenway et al. (private communication). Email: [kyle.greenway@mcgill.ca](mailto:kyle.greenway@mcgill.ca)  Questions 207-216, Big Five Inventory:  Rammstedt, B., & John, O. P. (2007). Measuring personality in one minute or less: A 10-item short version of the Big Five Inventory in English and German. *Journal of Research in Personality, 41*(1), 203–212.  *French version:*  Courtois R, Petot JM, Plaisant O, Allibe B, Lignier B, Réveillère C, Lecocq G, John O. Validation française du Big Five Inventory à 10 items (BFI-10) [Validation of the French version of the 10-item Big Five Inventory]. Encephale. 2020 Dec;46(6):455-462. French. doi: 10.1016/j.encep.2020.02.006. Epub 2020 Apr 21. PMID: 32331765. |
| ***Psychological items*** | |
| The following questions are about the frequency at which you had various psychological experiences.  Please do not include experiences that occurred only while under the influence of alcohol, drugs or medications that were not prescribed.  In the past 3 months, have you…  [Radio buttons] *Scale: 1=Never, 2=Sometimes, 3=Often, 4=Nearly Always*   1. … felt as if people seem to drop hints about you or say things with a double meaning? 2. … felt as if some people are not what they seem to be? 3. … felt that you are being persecuted in anyway? 4. … felt as if there is a conspiracy against you? 5. … felt that people look at you oddly because of your appearance? 6. … felt as if electrical devices such as computers can influence the way you think? 7. … felt as if the thoughts in your head are being taken away from you? 8. … felt as if the thoughts in your head are not your own? 9. … your thoughts been so vivid that you were worried other people would hear them? 10. … heard your thoughts being echoed back at you? 11. … felt as if you are under the control of some force or power other than yourself? 12. … felt as if a double has taken place of a family member, friend or acquaintance? 13. … heard voices when you are alone? 14. … heard voices talking to each other when you are alone? 15. … seen objects, people or animals that other people can't see?   ---   1. How many people are so close to you that you can count on them if you have great personal problems? (This may include people in the family.) 2. None 3. 1-2 4. 3-5 5. 6 or more 6. How much interest and concern do people show in what you do? 7. A lot 8. Some 9. Uncertain 10. Little 11. None 12. How easy is it to get practical help from others if you should need it? 13. Very easy 14. Easy 15. Uncertain 16. Difficult 17. Very difficult 18. In the past 2 weeks, have you used any of the following substances, and if so, how often did you use them? 19. Energy Drink (such as redbull, monster, etc.) 20. Alcohol 21. Cannabis (ex: marijuana, pot, hash, vape, etc.) 22. Amphetamines (ex: Speed) 23. Not prescribed psychostimulant medication such as Ritalin, Vyvanse, Adderall, etc. 24. Other (Specify)   [Radio buttons] *Scale: 1=Never, 2=Once or twice a week, 3=3 or more times a week but not every day, 4=Everyday, 5 = I prefer not to answer*   1. I am confirming that I completed this questionnaire in: 2. English 3. French | Questions 217-231, Current CAPE-15:  Capra, C., Kavanagh, D. J., Hides, L., & Scott, J. G. (2017). Current CAPE-15 : A measure of recent psychotic-like experiences and associated distress. *Early Intervention in Psychiatry*, *11*(5), 411‑417. <https://doi.org/10.1111/eip.12245>  *French version*:  Brenner, K., Schmitz, N., Pawliuk, N., Fathalli, F., Joober, R., Ciampi, A., & King, S. (2007). Validation of the English and French versions of the Community Assessment of Psychic Experiences (CAPE) with a Montreal community sample. *Schizophrenia Research*, *95*(1‑3), 86‑95. <https://doi.org/10.1016/j.schres.2007.06.017>  *Modification:*   - The prompt about drugs is adapted from the WHO Interview, as done in the QLSCD and Vincent’s Green/Screen Study.   Questions 232-234, Oslo Social Support Scale:  Kocalevent, R. D., Berg, L., Beutel, M. E., Hinz, A., Zenger, M., Härter, M., ... & Brähler, E. (2018). Social support in the general population: standardization of the Oslo social support scale (OSSS-3). *BMC psychology*, *6*, 1-8.  Question 235, substance use:  Adapted from:  Landry, M., Tremblay, J., Guyon, L., Bergeron, J., & Brunelle, N. (2004). La Grille de dépistage de la consommation problématique d’alcool et de drogues chez les adolescents et les adolescentes (DEP-ADO): développement et qualités psychométriques. *Drogues, santé et société*, *3*(1), 20-37.  Question 236:  Ad hoc |

**Daily diaries and digital phenotyping (n=100)**

| ***Well-being*** | |
| --- | --- |
| Please indicate how the following statements describe how you felt today.  [Radio buttons] *Scale: 1=Not at all, 2, 3, 4, 5, 6, 7=Very much*   1. Overall, I'm content with how my day went. 2. I was able to handle today's challenges well. 3. I am looking forward to tomorrow. 4. Today, I was able to concentrate and focus well. 5. Today, I felt connected to other people. 6. Today, I felt down or depressed. 7. Today, I had little interest or pleasure in doing things. 8. Today, I experienced physical discomfort/pain. 9. Today, it was difficult to cope with my emotions. 10. Today, I felt productive/useful. 11. Today, I felt sleepy. | Questions 1-10, well-being:  Fried, E. I., Proppert, R. K., & Rieble, C. L. (2023). Building an early warning system for depression: rationale, objectives, and methods of the WARN-D study. *Clinical psychology in Europe*, *5*(3), e10075.  Question 11, sleepiness:  Ad hoc |
| ***Sleep*** | |
| About your sleep…  [Dropdown menus] *0-24 hours* (1-hour intervals)*: 0, 15, 30, 45 minutes*   1. What time did you go you bed last night? 2. How long did it take for you to fall asleep last night? 3. At what time did you get out of bed today? | Questions 12-14, sleep schedule:  Adapted from:  Roenneberg, T., Wirz-Justice, A., & Merrow, M. (2003). Life between clocks: daily temporal patterns of human chronotypes. *Journal of biological rhythms*, *18*(1), 80-90. |
| ***Affect*** | |
| Please indicate how the following statements describe how you feel today.  [Radio buttons] *Scale: 1=Not at all, 2, 3, 4, 5, 6, 7=Very much*   1. Today, I felt happy. 2. Today, I felt relaxed. 3. Today, I felt energetic. 4. Today, I felt satisfied. 5. Today, I stressed. 6. Today, I anxious. 7. Today, I irritated. 8. Today, I down. | Questions 15-22:  Eisele, G., Lafit, G., Vachon, H., Kuppens, P., Houben, M., Myin-Germeys, I., & Viechtbauer, W. (2021). Affective structure, measurement invariance, and reliability across different experience sampling protocols. *Journal of Research in Personality*, 92, 104094. |
| ***Social interactions*** | |
| 1. Today, I spent time **virtually** (e.g., by phone, messaging, social media, video games) with (choose all that apply):   [Checklist]   - 1. Friend(s)   2. Romantic partner   3. Family   4. Classmate(s)/co-worker(s)   5. Acquaintance(s)/loose contact(s)   6. Stranger(s)   7. No one  1. [Skip if selected ‘No one’] Overall, I enjoyed spending time with them.   [Radio buttons] *Scale: 1=Not at all, 2, 3, 4, 5, 6, 7=Very much*   1. Today, I spent time **in person** with (choose all that apply):   [Checkboxes]   - 1. Friend(s)   2. Romantic partner   3. Family   4. Classmate(s)/co-worker(s)   5. Acquaintance(s)/loose contact(s)   6. Stranger(s)   7. Pet(s)   8. No one  1. [Skip if selected ‘No one’] Overall, I enjoyed spending time with them.   [Radio buttons] *Scale: 1=Not at all, 2, 3, 4, 5, 6, 7=Very much* | Questions 23-26:  Fried, E. I., Proppert, R. K., & Rieble, C. L. (2023). Building an early warning system for depression: rationale, objectives, and methods of the WARN-D study. *Clinical psychology in Europe*, *5*(3), e10075.  *Modifications:*   - The original item was about in-person interactions. An ad-hoc version was created here to focus on virtual interactions. - The follow-up question about enjoyment was modified for clarity. The original question was: “I enjoyed my company”. - The original item was about current interactions and was modified to capture past-day interactions (“Today, …”). |
| ***Daily events*** | |
| 1. My most negative event/experience today belongs to the category (choose all that apply):   [Checkboxes]   - 1. My physical wellbeing   2. My emotional/mental wellbeing   3. Finances   4. Education/work   5. Home/living situation   6. Leisure/hobby/exercise   7. Love life   8. Social life   9. Relationship with family   10. Experiences of friends/family   11. Societal/political  1. This event/experience was:   [Radio buttons] *Scale:* *1=Not at all negative, 2, 3, 4, 5, 6, 7=Very negative*   1. My most positive event/experience today belongs to the category (choose all that apply):   [Checkboxes]   - 1. My physical wellbeing   2. My emotional/mental wellbeing   3. Finances   4. Education/work   5. Home/living situation   6. Leisure/hobby/exercise   7. Love life   8. Social life   9. Relationship with family   10. Experiences of friends/family   11. Societal/political  1. This event/experience was:   [Radio buttons] *Scale:* *1=Not at all positive, 2, 3, 4, 5, 6, 7=Very positive* | Questions 27-30:  Fried, E. I., Proppert, R. K., & Rieble, C. L. (2023). Building an early warning system for depression: rationale, objectives, and methods of the WARN-D study. *Clinical psychology in Europe*, *5*(3), e10075. |
| ***Gaming behaviors*** | |
| 1. Have you played video games today?   [Radio buttons] *0=No, 1=Yes*  [If answered ‘Yes’ for past-day video gaming:]  Please indicate, approximately, how much time you spent playing during each of the following periods:  [Radio buttons] *Scale: 0=None, 1=Less than 1 hour, 2=1-2 hours, 3=3-4 hours, 4=5-6 hours*   1. Between 6 AM and noon 2. Between noon and 6 PM 3. Between 6 PM and midnight 4. Between midnight and 6 AM (of the next day)   [If answered ‘Yes’ for past-day video gaming:]   1. *(embargoed: questionnaire about in-game-positive and negative interactions)* | Questions 31-35, gaming schedule:  Allen, J. J. (2020). Gaming as psychologically nutritious: Does need satisfaction in video games contribute to daily well-being beyond need satisfaction in the real world? (Doctor of Philosophy, Iowa State University). Iowa State University. <https://doi.org/10.31274/etd-20200902-2>  They adapted these items from the General Media Habits Questionnaire; Gentile et al., 2004.  Questions 36, in-game positive and negative interactions (10 items):  Hygen, B. et al. (private communication) |
| ***Gaming experiences: Need satisfaction*** *(skip if said ‘No’ to past-day video gaming)* | |
| Please indicate the degree to which each statement characterizes your experiences of gaming in general today.  Throughout my experiences playing games today…  [Radio buttons] *Scale: 1=Strongly disagree, 2, 3, 4=Neither agree nor disagree, 5, 6, 7=Strongly agree*   1. I could make choices regarding how to play the games. 2. I could play the games in the way I wanted. 3. I could direct my own play experience in the games. 4. I felt forced to take certain actions in the games. 5. Many actions in the games were boring. 6. I often found myself wishing I could do something else within the games. 7. I felt I was getting better at playing the games. 8. I felt that I made progress while playing the games. 9. I felt a sense of achievement while playing the games. 10. I often felt that I lacked the skills necessary for the games. 11. I kept failing to accomplish what I wanted to while playing the games. 12. I felt disappointed with my performance in the games. 13. I felt I formed relationships with other players and/or characters in the games. 14. Engaging with the games, I felt a connection to others, virtual or real. 15. I felt that other players and/or characters in the games cared about me. 16. Interactions with other players and/or characters in the games felt toxic to me. 17. The community or virtual world in the games made me feel unwelcome. 18. Others in the games were unfriendly towards me. | Questions 37-54, Basic Needs in Games Scale:  Ballou, N., Denisova, A., Ryan, R., Rigby, C. S., & Deterding, S. (2024). The Basic Needs in Games Scale (BANGS): A new tool for investigating positive and negative video game experiences. *International Journal of Human-Computer Studies*, *188*, 103289 |
| ***Immersion*** *(skip if said ‘No’ to past-day video gaming)* | |
| Throughout my experiences playing games today…  [Radio buttons] *Scale: 1=Strongly disagree, 2, 3, 4=Neither agree nor disagree, 5, 6, 7=Strongly agree*   1. I was no longer aware of my surroundings while I was playing. 2. I was immersed in the game. 3. I was fully focused on the game. | Questions 55-57, immersion subscale of the Player Experience Inventory:  Vanden Abeele, V., Spiel, K., Nacke, L., Johnson, D., & Gerling, K. (2020). Development and validation of the player experience inventory: A scale to measure player experiences at the level of functional and psychosocial consequences. *International Journal of Human-Computer Studies*, *135*, 102370. |
| ***Prosocial behaviors*** | |
| 1. [skip if said ‘No’ to past-day video gaming)] Today*,* have you helped or supported a non-playable character in a video game?   [Radio buttons] *0 = No, 1 = Yes*   1. [skip if said ‘No’ to previous question] How much effort was involved in helping or supporting this/these character(s)?   [Radio buttons] *Scale: 1=Not at all, 2, 3, 4, 5, 6, 7=Very much*   1. [skip if said ‘No’ to past-day video gaming)] Today*,* have you helped or supported a human player in a video game?   [Radio buttons] *0 = No, 1 = Yes*   1. [skip if said ‘No’ to previous question] How much effort was involved in helping or supporting this/these player(s)?   [Radio buttons] *Scale: 1=Not at all, 2, 3, 4, 5, 6, 7=Very much*   1. Today, have you helped or supported someone outside of video games?   [Radio buttons] *0 = No, 1 = Yes*   1. [skip if said ‘No’ to previous question] How much effort was involved in helping or supporting this person?   [Radio buttons] *Scale: 1=Not at all, 2, 3, 4, 5, 6, 7=Very much* | Questions 58-63:  Pronizius, E., Forbes, P. A., Feneberg, A. C., Miculescu, B., Nater, U. M., Piperno, G., ... & Lamm, C. (2024). Everyday helping is associated with enhanced mood but greater stress when it is more effortful. *Scientific Reports*, *14*(1), 24120.  *Modifications:*   - The original question was expanded to differentiate in-game and off-game prosocial behaviors. - The response options for effort were originally on a 0-100 scale but were replaced with a 7-point scale for consistency with previous items. |
| ***Streaming behaviors*** | |
| 1. Have you streamed or watched a video game stream today?   [Radio buttons] *0=No, 1=Yes*  [If answered ‘Yes’ for past-day streaming:]  Please indicate, approximately, how much time you spent streaming or watching video game streams during each of the following periods:  [Radio buttons] *Scale: 0=None, 1=Less than 1 hour, 2=1-2 hours, 3=3-4 hours, 4=5-6 hours*   1. Between 6 AM and noon 2. Between noon and 6 PM 3. Between 6 PM and midnight 4. Between midnight and 6 AM (of the next day) | Idem as gaming questionnaires above, adapted from streaming. |
| ***Streaming experiences: Need satisfaction*** *(skip if said ‘No’ to past-day streaming)* | |
| Please indicate the degree to which each statement characterizes your experiences of streaming and/or watching video game streams in general today.  Throughout my experiences streaming and/or watching video game streams today…  [Radio buttons] *Scale: 1=Strongly disagree, 2, 3, 4=Neither agree nor disagree, 5, 6, 7=Strongly agree*   1. I could make choices regarding what happened in the stream. 2. I could stream or watch the streams in the way I wanted. 3. I could direct my own experience in the streams. 4. I felt forced to take certain actions in the streams. 5. Many actions in the streams were boring. 6. I often found myself wishing I could do something else within the streams. 7. I felt I was getting better at playing the games while streaming or watching the streams of the games. 8. I felt that I made progress while streaming or watching the streams of the games. 9. I felt a sense of achievement while streaming or watching the streams of the games. 10. I often felt that I lacked the skills necessary for the games while streaming or watching the streams of the games. 11. I kept failing to accomplish what I wanted to while streaming or watching the streams. 12. I felt disappointed with my performance in the games while streaming or watching the streams of the games. 13. I felt I formed relationships with other streamers, stream viewers and/or characters in the games. 14. Engaging with the streams, I felt a connection to others, virtual or real. 15. I felt that other streamers, stream viewers and/or characters in the games cared about me. 16. Interactions with other streamers, stream viewers and/or characters in the games felt toxic to me. 17. The community or virtual world in the streams made me feel unwelcome. 18. Others in the streams were unfriendly towards me. | Idem as gaming questionnaires above, adapted from streaming. |
| ***Immersion*** *(skip if said ‘No’ to past-day streaming)* | |
| Throughout my experiences streaming games today…  [Radio buttons] *Scale: 1=Strongly disagree, 2, 3, 4=Neither agree nor disagree, 5, 6, 7=Strongly agree*   1. I was no longer aware of my surroundings while I was streaming or watching a video game stream. 2. I was immersed in the stream. 3. I was fully focused on the stream. | Idem as gaming questionnaires above, adapted from streaming. |
| 1. Todays was the last survey of the study. A thousand thanks for your participation! As a last question, we would be interested in having your feedback on the content of the questionnaire.   Was there anything important that seemed to be missing from the questions? Is there anything else you think we should consider in this research?  [Large textbox] | |
