# Supplementary material for: Understanding the game experiences and mental health of youth: protocol for the Game-in-Action Quebec cohort study
Source: BMJ Open. 2025 Sep 1;15(9):e103685. doi: 10.1136/bmjopen-2025-103685 (PMC12406825; doi:10.1136/bmjopen-2025-103685)

PEER REVIEW HISTORY

BMJ Open publishes all reviews undertaken for accepted manuscripts. Reviewers are asked to complete a checklist review form and are provided with free text boxes to elaborate on their assessment. These free text comments are reproduced below.

ARTICLE DETAILS

Title (Provisional)

Understanding the Game Experiences and Mental Health of Youth: Protocol for the Game-in-Action Quebec Cohort Study

Authors

Paquin, Vincent; Raugh, Ian; Davies, Megan; Lepage, Martin; Lavigne, Katie; Dion, Jacinthe; Abdel Baki, Amal; Shah, Jai; Iyer, Srividya; Ferrari, Manuela

---

VERSION 1 - REVIEW

**Reviewer**	**1**
**Name**	**Strojny, Paweł**
**Affiliation**	**Jagiellonian University**
**Date**	**10-Jun-2025**
**COI**	**None**

Thank you for the opportunity to review this study protocol. The topic is timely and relevant, particularly in light of increasing interest in digital phenotyping and experience sampling in the context of gaming behavior. However, several aspects of the manuscript raise concerns—both minor and more substantial—regarding clarity, methodological coherence, and the broader rationale for publishing the protocol at this stage.

Minor issues:

Page 4, Line 12: The current presentation of gender differences in gaming popularity is difficult to interpret. Please consider expressing these differences with a common metric—e.g., the proportion of males and females playing more than 12 hours per week—to allow for easier comparison.

Page 12, Line 46: Referring to 16-year-olds as "adults" may be administratively justified, but it feels counterintuitive from both a developmental and legal perspective. A brief clarification might help.

Page 13, Line 6: I am unfamiliar with the Beiwe application. I was unable to locate it in the Android app store—is access restricted? Based on my experience, using unfamiliar or hard-

to-access apps in ESM studies poses a considerable barrier to participant engagement and retention.

Major issues:

Page 12, Line 54: Objective 3 concerns not only gender but also ethnoracial and mental health-related differences. However, the described stratification and subsampling procedure addresses gender proportions only. I strongly recommend that the authors consider a more comprehensive sampling strategy to ensure the feasibility of testing their stated hypotheses across all subgroups.

Use of geolocation (time spent at home): While the variable itself may hold value, I found no theoretical or empirical justification for its inclusion in the Introduction. The rationale should be clarified—why this specific measure was chosen and how it is hypothesized to relate to gaming behavior or mental health outcomes.

General comment:

I understand and fully support that BMJ Open allows the publication of study protocols, which is in line with important goals of the journal: increasing transparency, promoting collaboration and avoiding unnecessary duplication of research work.

Nevertheless, it is hard for me not to notice that publishing a protocol at a time when the majority of data is likely already collected – or when a significant part of it (the digital phenotyping component) will be collected long after the baseline data has been measured – undermines the value of the declared transparency in practice. There is a risk that data collected with a significant delay from the baseline measurement will only reflect the original state of the participants to a limited extent – their life situation may have changed significantly, which potentially distorts the conclusions from the analyses.

In this context, the publication of the protocol does not fully meet the goal of transparency of research plans before their implementation. It is therefore difficult not to get the impression that we are dealing with a form of retrospective justification rather than a real pre-registration protocol. In my opinion, it is worth the editors to consider whether such cases really fit within the declared mission of publishing study protocols.

**Reviewer**	**2**
**Name**	**Zhu, Shimin**
**Affiliation**	**The Hong Kong Polytechnic University**
**Date**	**17-Jun-2025**
**COI**	**None**

This protocol presents a valuable, methodologically innovative study addressing an important topic. To enhance rigor and clarity, the authors should:

1) Provide more detail on sampling strategies, measurement validation, and confounder control. The use of convenience sampling limited to Quebec youth is acknowledged as a limitation but could be better addressed by describing any strategies to maximize sample diversity (e.g., recruitment across different socioeconomic strata, urban vs. rural). This would strengthen external validity.

2) While validated self-report measures are used, the protocol could be improved by specifying which scales are employed, their psychometric properties in similar populations, and how measurement invariance over time will be assessed.

3) For novel or adapted measures (e.g., streaming experiences), pilot testing or validation plans should be described.

4) Consider including power analysis details to justify the sample size of 1000 for the survey and 100 for digital phenotyping, ensuring sufficient power to detect hypothesized effects.

5) Elaborate on digital phenotyping procedures and ethical safeguards.

6) Add visual aids to clarify the theoretical framework and study design.

Addressing these specific points will strengthen the manuscript's scientific rigor and accessibility, increasing its impact in the field.

VERSION 1 - AUTHOR RESPONSE

Reviewer: 1

R1.1. Thank you for the opportunity to review this study protocol. The topic is timely and relevant, particularly in light of increasing interest in digital phenotyping and experience sampling in the context of gaming behavior. However, several aspects of the manuscript raise concerns—both minor and more substantial—regarding clarity, methodological coherence, and the broader rationale for publishing the protocol at this stage.

RESPONSE: We thank the reviewer for their feedback which was helped to improve the manuscript, and we are glad that they found the digital phenotyping approach in the context of gaming behavior to be timely and relevant.

R1.2. Page 4, Line 12: The current presentation of gender differences in gaming popularity is difficult to interpret. Please consider expressing these differences with a common metric—e.g., the proportion of males and females playing more than 12 hours per week—to allow for easier comparison.

RESPONSE: This passage was revised to present gender differences with a common metric:

> **"In Canada, the average video gaming time is 6.5 hours/week among female young adults and 10.2 hours/week among male young adults, in line with global trends [4,5]." (p. 4, paragraph 1)**

R1.3. Page 12, Line 46: Referring to 16-year-olds as "adults" may be administratively justified, but it feels counterintuitive from both a developmental and legal perspective. A brief clarification might help.

RESPONSE: We agree that referring to 16-year-olds as "adults" may be unusual. We use the term "adolescents" to refer to 16- and 17-year-olds:

> **"For the cross-sectional questionnaire, we will recruit 1000 adolescent and adult gamers from the general population" (p. 12, paragraph 1)**

R1.4. Page 13, Line 6: I am unfamiliar with the Beiwe application. I was unable to locate it in the Android app store—is access restricted? Based on my experience, using unfamiliar or hard-to-access apps in ESM studies poses a considerable barrier to participant engagement and retention.

RESPONSE: The Beiwe platform, developed by the Onnela Lab at Harvard T.H. Chan School of Public Health, is a research-focused mobile application designed for digital phenotyping and ecological momentary assessment. It is available for public download via the iOS app store, whereas for Android phones, the app must be distributed directly to participants through custom installation links provided by the research team.

One key reason we selected Beiwe for this study is that it allows us to store all collected data on secure servers located in Quebec. This ensures compliance with new provincial legislation concerning data sovereignty and the protection of personal information (e.g., Law 25).

We acknowledge that the need for a manual installation process can introduce challenges. However, in our study, we plan to provide step-by-step guidance and technical support to all participants during onboarding to minimize these barriers.

Additional information is now provided to indicate how the app can be accessed and the kind of support provided.

> **"During the meeting … participants will receive assistance to install the Beiwe application on their smartphone." (p. 14, paragraph 1)**
>
> **"It [the app] can be downloaded from the Centre Axel website (https://beiwe.centreaxel2.com/download) for Android phones and from the App Store for iOS phones." (p. 14, paragraph 3)**

R1.5. Major issues: Page 12, Line 54: Objective 3 concerns not only gender but also ethnoracial and mental health-related differences. However, the described stratification and subsampling procedure addresses gender proportions only. I strongly recommend that the authors consider a more comprehensive sampling strategy to ensure the feasibility of testing their stated hypotheses across all subgroups.

RESPONSE: As the reviewer noted, stratification of participants in the digital phenotyping sample will only be by gender. This decision was made early in the project's conception to account for gender differences in gaming practices and toxicity.

It is only later that we opted to add other identity labels in Objective 3 (ethnoracial groups and history of mental health problem) following consultations with our advisory committee. Currently, it is not possible to modify the stratification strategy since this would require an amendment to ethics that we cannot obtain immediately, and BMJ Open does not permit modifications of the protocol during the review process.

However, we wish to highlight that we are using other strategies to increase sample diversity during recruitment of the cross-sectional sample. We now provide additional information about this:

> **"To maximize sample diversity, we have contacted several community organizations with missions related to mental health, LGBTQ+ communities, and the well-being of immigrant and racialized youths, as well as university student groups and online communities with game-adjacent interests (e.g., roleplay games, cosplay, geek) across the province of Quebec who circulated the advertisement through their networks." (p. 13, paragraph 2)**

Recruitment is still ongoing, but preliminary data at time of writing (n=738) indicates reasonable group sizes for analyses, e.g., ethnoracial groups, Asian 11%, Black 10%, White 67%; history of mental health condition, Yes 19%, No 78%.

We now discuss sampling adequacy as a limitation of the study and area for future research:

> **"While the present study aims to recruit participants from diverse backgrounds, it is likely that convenience sampling across groups will not be sufficient to uncover all potential group-based differences. A future direction for research will thus be to incorporate more targeted sampling strategies to better capture differences in gaming experiences related to sociocultural and personal circumstances, such as ethnoracial groups, LGBTQ+ identities, mental health, and disabilities." (p. 24, paragraph 1)**

R1.6. Use of geolocation (time spent at home): While the variable itself may hold value, I found no theoretical or empirical justification for its inclusion in the Introduction. The rationale should be clarified—why this specific measure was chosen and how it is hypothesized to relate to gaming behavior or mental health outcomes.

RESPONSE: We now provide the justification for the inclusion of geolocation in the Introduction.

> **"Digital phenotyping, which involves ambulatory collection of active (e.g., surveys) and passive (e.g., geolocation) data, may provide indirect markers of gaming disorder [37]; notably, greater time spent at home according to geolocation has been associated with depressive symptoms [38] and could hypothetically index reduced mobility as a consequence of prolonged gaming sessions." (p. 7, paragraph 1)**

R1.7. General comment: I understand and fully support that BMJ Open allows the publication of study protocols, which is in line with important goals of the journal: increasing

transparency, promoting collaboration and avoiding unnecessary duplication of research work.

Nevertheless, it is hard for me not to notice that publishing a protocol at a time when the majority of data is likely already collected – or when a significant part of it (the digital phenotyping component) will be collected long after the baseline data has been measured – undermines the value of the declared transparency in practice. There is a risk that data collected with a significant delay from the baseline measurement will only reflect the original state of the participants to a limited extent – their life situation may have changed significantly, which potentially distorts the conclusions from the analyses.

In this context, the publication of the protocol does not fully meet the goal of transparency of research plans before their implementation. It is therefore difficult not to get the impression that we are dealing with a form of retrospective justification rather than a real pre-registration protocol. In my opinion, it is worth the editors to consider whether such cases really fit within the declared mission of publishing study protocols.

RESPONSE: BMJ Open was selected as the publication venue because it allows for the publication of study protocols after data collection has begun, provided that data collection is not complete (https://bmjopen.bmj.com/pages/authors/#protocols).

Our team has followed this guideline carefully. Recruitment for the cross-sectional sample is still ongoing. Importantly, we have not started data collection for digital phenotyping yet, and this data is required for most of the analyses indicated in the research questions (Table 1 of the manuscript).

The delay between the cross-sectional survey and digital phenotyping indeed poses challenges for the interpretation of findings, given the possibility that participants' situation will evolve over time. We will consider the impact of the delay in the interpretation of the eventual study findings. For example, the delay could attenuate the strength of the association between Modified Fandom Functions Scale – Belonging (cross-sectional) and daily well-being (digital phenotyping) in Objective 1, given that the experience of belonging in game communities may change with time. On the other hand, many of the research questions will be examined within the digital phenotyping data only (e.g., the association between daily Basic Needs in Games and daily well-being, Objective 1) and thus will involve a shorter timeframe. Finally, the delay may be less impactful for cross-sectional measures that are relatively stable over time, such as the personal propensity for immersion (Modified Tellegen Absorption Scale; Objective 2) and gender and ethnoracial groups (Objective 3).

Additionally, we wish to point out that the maximum time gap between the cross-sectional assessment and the start of digital phenotyping will be no more than 12 months, with most cases falling under six months. We recognize that life circumstances can change rapidly, which is why continuous and real-time data collection through digital phenotyping will be valuable for capturing such dynamic shifts.

We are committed to ongoing transparency and will ensure that any deviations from the original protocol are clearly reported in subsequent publications. Sharing our protocol at this stage still serves the broader goals of transparency and openness, especially by documenting our methodology and decision-making prior to analysis.

Reviewer: 2

R2.1. This protocol presents a valuable, methodologically innovative study addressing an important topic.

RESPONSE: We thank the reviewer for their positive feedback and suggestions which have helped to improve the manuscript.

R2.2. To enhance rigor and clarity, the authors should: 1) Provide more detail on sampling strategies, measurement validation, and confounder control. The use of convenience sampling limited to Quebec youth is acknowledged as a limitation but could be better addressed by describing any strategies to maximize sample diversity (e.g., recruitment across different socioeconomic strata, urban vs. rural). This would strengthen external validity.

RESPONSE: We appreciate the suggestion to provide greater detail on sampling strategies, measurement validation, and confounder control, and we have revised the manuscript accordingly to enhance rigor and clarity. We now provide additional information on strategies used to maximize sample diversity.

> **"To maximize sample diversity, we have contacted several community organizations with missions related to mental health, LGBTQ+ communities, and the well-being of immigrant and racialized youths, as well as university student groups and online communities with game-adjacent interests (e.g., roleplay games, cosplay, geek) across the province of Quebec who circulated the advertisement through their networks." (p. 13, paragraph 2)**

For confounding control, we mention the following:

> **"We will analyze cross-sectional outcomes using generalized linear models adjusted for relevant socio-demographic characteristics (e.g., sex, educational attainment, and occupational status)." (p. 21, paragraph 2)**

> **"To parse out within- and between-person associations between longitudinal repeated measures (e.g., the association between daily psychological need satisfaction and daily wellbeing), we will apply person- and mean-centering to the predictors; this approach accounts for time-invariant, unmeasured individual factors that could confound associations between the predictor and outcome" (p. 22, paragraph 1)**

There is no specific strategy to oversample disadvantaged socioeconomic groups or rural populations. We agree that these groups may be underrepresented in the final sample and have modified the analysis section to account for this potential limitation:

> **"Characteristics of the analytic samples (cross-sectional and longitudinal) will be described to identify the potential underrepresentation of groups (e.g., gender, ethnoracial, socioeconomic, or rural vs. urban groups) that may limit the generalizability of findings." (p. 21, paragraph 2)**

2) While validated self-report measures are used, the protocol could be improved by specifying which scales are employed, their psychometric properties in similar populations, and how measurement invariance over time will be assessed.

RESPONSE: We now provide additional information on the scales employed and, when available, their psychometric properties. For example, we added the name of the scale and properties in the following passage:

> **"The use of games to recuperate from stress will be evaluated using 4 items adapted from the Recovery Experience Questionnaire (e.g., "When I play video or computer games I forget about work") rated on a scale of 1=does not apply at all to 5=does fully apply [58,59]. The full questionnaire, tested in a sample of young adults in Germany, included four subscales (total 16 items), psychological detachment α = .78, relaxation: α = .70, mastery: α = .76, control: α = .77, which were positively associated with greater time spent on gaming [58]." (p. 16, paragraph 1)**

We now comment on the assessment of psychometric properties for longitudinal measures:

> **"Using lavaan [84], we will examine the factor structure and internal consistency of ad-hoc or modified questionnaires and, for analyses of longitudinal changes over time, we will also test for longitudinal measurement invariance [e.g., 85]." (p. 21, paragraph 2)**

3) For novel or adapted measures (e.g., streaming experiences), pilot testing or validation plans should be described.

We now specify that the streaming measures were pilot-tested for feedback:

> **"Since these modified questionnaires have not been validated, they were pilot-tested by the research team and Youth Advisory (see Patient and public involvement, below), and their psychometric properties will be examined in the present study (see Analyses, below)." (p. 17, paragraph 2)**

We also comment on the assessment of psychometric properties for novel or adapted measures:

> **"Using lavaan [84], we will examine the factor structure and internal consistency of ad-hoc or modified questionnaires and, for analyses of longitudinal changes over time, we will also test for longitudinal measurement invariance [e.g., 85]." (p. 21, paragraph 2)**

4) Consider including power analysis details to justify the sample size of 1000 for the survey and 100 for digital phenotyping, ensuring sufficient power to detect hypothesized effects.

RESPONSE: We now provide additional information about the power analyses.

> **"Given the variety of objectives and analyses planned, we established the sample size based on two basic scenarios: a linear regression of one cross-sectional**

variable over another (n=1000) and a generalized linear mixed model where one daily digital phenotyping variable is regressed over another (n=100). For the cross-sectional scenario, we conducted the power analyses in G*Power, version 3.1.9.7. We calculated that a sample of n=1000 will be sufficient to detect a linear regression slope of β=0.09, assuming a type I error rate of 0.05 (two-tailed) and statistical power of 85%. This would be sufficient to evaluate, for example, a weak but significant association between greater belonging and lower levels of psychotic-like experiences (Objective 1) or a greater propensity for immersion and greater psychological functions of games (Objective 2). For the digital phenotyping component, we used the sample size calculator developed by Barnett and colleagues [54]: https://onnela-lab.shinyapps.io/digital_phenotyping_sample_size_calculator/. We found that a sample size of 100 persons with daily measures over 2 weeks will provide statistical power of 93% to detect effect sizes of β=0.07 with a type I error rate of 0.05, assuming 50% missing data and random-effect variance of 1.1. These parameters would be sufficient to examine the association between daily needs satisfaction in games and daily well-being (Objective 1) or the association between daily immersion and daily well-being (Objective 2). This statistical power estimation also applies to passive sensor data (e.g., geolocation) transformed into daily features (e.g., daily time spent at home).” (p. 12, paragraph 2)

5) Elaborate on digital phenotyping procedures and ethical safeguards.

RESPONSE: We now provide additional information on the digital phenotyping procedures and ethical safeguards.

> **“It [the app] can be downloaded from the Centre Axel website (https://beiwe.centreaxel2.com/download) for Android phones and from the App Store for iOS phones. Participants will be instructed to let the application run in the background throughout the 2-week follow-up period.” (p. 14, paragraph 3)**
>
> **“Apps commonly used by adolescents and adults frequently collect and upload on the internet their geolocation data, often without users’ full awareness [88]. For the current research, we are emphasizing this aspect of the digital phenotyping component in the consent form to ensure participants’ full awareness and consent of the procedure. Data will be stored on secure servers located in Montreal, Canada that are fully compliant with Quebec regulations on data privacy.” (p. 23, paragraph 2)**
>
> **“We also acknowledge that some questions may trigger discomfort or distress in participants. Participants will be informed that they can contact the research team at any point to receive assistance as needed, including immediate support and orientation to appropriate psychological or social services in the community.” (p. 23, paragraph 3)**

6) Add visual aids to clarify the theoretical framework and study design.
Addressing these specific points will strengthen the manuscript’s scientific rigor and accessibility, increasing its impact in the field.

RESPONSE: We added a figure (Figure 1) that summarizes the study design and theoretical framework:

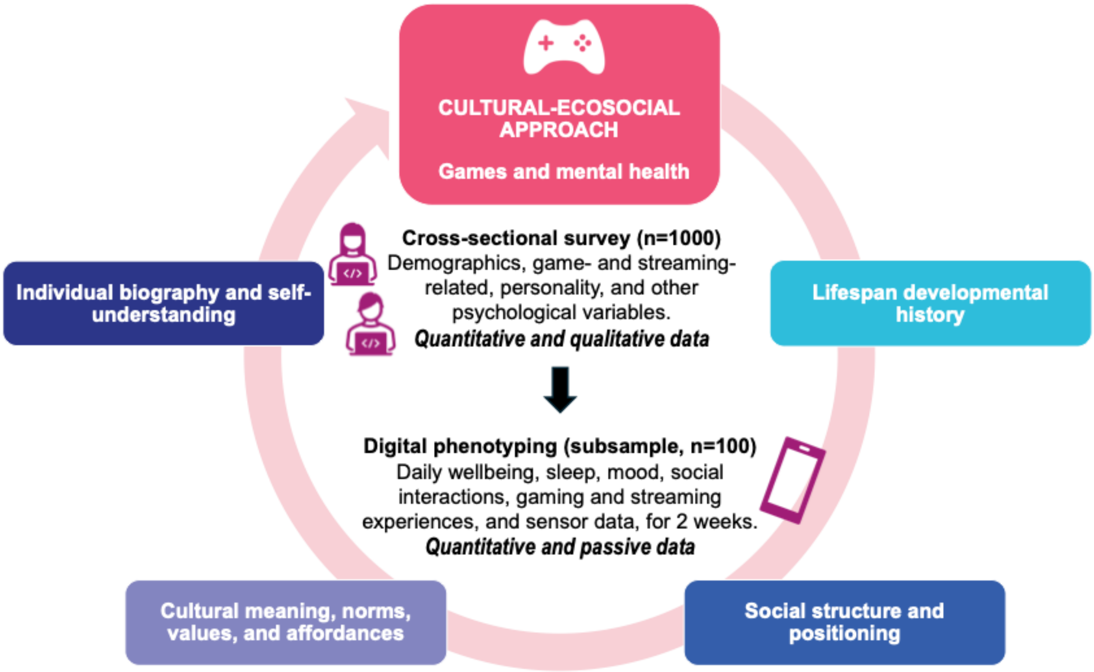

Supplement: Supplementary file 2 [file bmjopen-2025-103685.reviewer_comments.pdf]
